# Weight loss since early adulthood, later life risk of fracture hospitalizations, and bone mineral density: a prospective cohort study of 0.5 million Chinese adults

**DOI:** 10.1007/s11657-020-00734-3

**Published:** 2020-04-19

**Authors:** Zewei Shen, Canqing Yu, Yu Guo, Zheng Bian, Yuxia Wei, Huaidong Du, Ling Yang, Yiping Chen, Yulian Gao, Xukui Zhang, Junshi Chen, Zhengming Chen, Jun Lv, Liming Li, on behalf of the China Kadoorie Biobank Collaborative Group

**Affiliations:** 1 Department of Epidemiology and Biostatistics, School of Public Health, Peking University Health Science Center, Beijing, China; 2 Chinese Academy of Medical Sciences, Beijing, China; 3 Clinical Trial Service Unit and Epidemiological Studies Unit (CTSU), Nuffield Department of Population Health, University of Oxford, Oxford, UK; 4 Medical Research Council Population Health Research Unit (MRC PHRU), Nuffield Department of Population Health, University of Oxford, Oxford, UK; 5 Huixian Center for Disease Control and Prevention, Huixian, Henan China; 6 China National Center for Food Safety Risk Assessment, Beijing, China; 7 Key Laboratory of Molecular Cardiovascular Sciences (Peking University), Ministry of Education, Beijing, China; 8 Peking University Institute of Environmental Medicine, Beijing, China

**Keywords:** Weight loss, Fracture, Bone mineral density, Prospective cohort study

## Abstract

***Summary*:**

In a Chinese population from both urban and rural areas, weight loss of ≥ 5 kg from early adulthood to midlife was associated with a higher risk of hip fracture and lower BMD in later life.

**Introduction:**

This study investigates the association of the long-term weight loss from young adulthood through the middle ages with the subsequent 10-year risk of hospitalized fracture and calcaneus bone mineral density (BMD).

**Methods:**

China Kadoorie Biobank (CKB) was established during 2004–2008 in ten areas across China. Weight at age 25 years was self-reported at baseline, and weight at baseline and resurvey was measured by the calibrated equipment. Outcomes were hospitalized fracture during follow-up and calcaneus BMD measured at resurvey. Analysis for fracture risk included 411,812 participants who were free of fracture in the last 5 years before baseline, cancer, or stroke at any time before baseline. Analysis for BMD included 21,453 participants who participated in the resurvey of 2013–2014 with the same exclusion criteria as above.

**Results:**

The mean age was 50.8 at baseline and 58.4 at resurvey. Median weight change from age 25 to baseline was 4.4 kg, with 20.7% losing weight and 58.5% gaining weight. During a median follow-up of 10.1 years, we documented 13,065 cases of first diagnosed fracture hospitalizations, including 1222 hip fracture. Compared with participants whose weight was stable (± 2.4 kg), the adjusted hazard ratios (95% CIs) for those with weight loss of ≥ 5.0 kg from age 25 to baseline was 1.39 (1.17 to 1.66) for hip fracture. Weight loss was not associated with fracture risk at other sites. Those with weight loss from age 25 to resurvey had the lowest BMD measures, with β (95% CIs) of − 4.52 (− 5.08 to − 3.96) for broadband ultrasound attenuation (BUA), − 4.83 (− 6.98, − 2.67) for speed of sound (SOS), and − 4.36 (− 5.22, − 3.49) for stiffness index (SI).

**Conclusions:**

Weight loss from early adulthood to midlife was associated with a higher risk of hip fracture and lower BMD in later life.

**Electronic supplementary material:**

The online version of this article (10.1007/s11657-020-00734-3) contains supplementary material, which is available to authorized users.

## Introduction

The prevalence of weight loss attempts, for which concerns about health and appearance are the most common motivators, has been increasing among adults worldwide in the last decades [1]. Such weight loss intention is even stronger in younger and middle-aged adults [2]. Previous studies mainly focused on the health effects of weight gain across adulthood and had associated it with the significantly increased risk of major chronic diseases and decreased odds of healthy aging [3]. However, much less attention has been devoted to the potential influence of weight loss since early adulthood on health outcomes later in life, such as bone health, a growing concern as the population ages.

There were studies that have related increased risk of hip fracture [4–7] and decreased BMD [8–10] to weight loss since middle and late adulthood or during the postmenopausal years. Only a few prospective studies conducted in the Western countries and mostly in women have reported that weight loss since early adulthood was associated with a higher risk of hip fracture [11–13] and lower BMD [14, 15] in late adulthood. The association of weight loss with the fracture may differ by fracture site. However, the findings of the association between weight loss and fractures at sites other than hip, such as upper limb [6, 7] and spine [7, 16], were inconsistent. Also, these three available studies were conducted in either postmenopausal women [6, 7] or men ≥ 65 years old [16] and to address the association of weight loss after baseline with the risk of fracture at various body sites during the follow-up. Despite the evidence available in the Western populations, the findings might not be fully applied to the Asian populations including Chinese. Compared with Western populations, Asian populations exhibit lower body weights but higher prevalence of weight loss attempts because of local culture and norms [17]. Thus, the weight loss from overweight or normal weight occurs frequently in the Asian populations, compared with the weight loss from morbid obesity or obesity in the Western populations.

In the present China Kadoorie Biobank (CKB) of 0.5 million adults, we first examined the association between the long-term weight loss from young adulthood through the middle ages and the subsequent 10-year risk of hospitalized fracture. Second, in a sub-sample of about 5% of CKB participants, we used calcaneus BMD as a measure of bone health and examined its association with the long-term weight loss. We further investigated which of the stages during adulthood in which weight loss occurs contributed most to the BMD.

## Materials and methods

### Study population

The CKB is a prospective cohort study of 512,715 participants aged 30–79 years from 10 geographically diverse survey sites across China. Participants were enrolled between June 2004 and July 2008 and have been followed up ever since for morbidity and mortality. Periodic resurveys are conducted every 4–5 years and cover about 5% (~ 25,000) of the surviving participants. The first resurvey was conducted in 2008 and the second one in 2013–2014. Further details of the CKB study have been described elsewhere [18, 19]. In the present study, we used data from baseline, follow-up until 2016, and the second resurvey (hereinafter abbreviated to “resurvey”).

Both the Ethics Review Committee of the Chinese Center for Disease Control and Prevention (Beijing, China) and the Oxford Tropical Research Ethics Committee, University of Oxford (UK), have approved the CKB study. All participants signed informed consent forms before joining the study.

### Assessment of exposure and covariates

At both baseline and resurvey, participants were interviewed with a laptop-based electronic questionnaire, including information on sociodemographic status (age, sex, education, occupation, household income, and marital status), lifestyle (tobacco smoking, alcohol consumption, dietary habits, and physical activity), personal health and medical history (cancer, heart disease, stroke, diabetes, chronic obstructive pulmonary disease (COPD), fracture, and self-rated health), and female menopausal status.

Anthropometric measurements, including standing height and weight, were taken by trained personnel following standardized protocols and using the calibrated equipment. BMI was calculated as the weight in kilograms divided by the square of the height in meters (kg/m^2^). A stepwise on-site testing of plasma glucose level was undertaken using the SureStep Plus meter (LifeScan; Milpitas, CA, USA). Prevalent diabetes was defined as a measured fasting blood glucose ≥ 7.0 mmol/L, a measured random blood glucose ≥ 11.1 mmol/L, or self-reported previous diagnosis of diabetes. Measurements of forced expiratory volume in 1 s (FEV1) and forced vital capacity (FVC) were made using the handheld Micro Spirometer (MS01; CareFusion UK Ltd., Basingstoke, UK). Prevalent COPD was defined as FEV_1_/FVC < 70% or self-reported previous diagnosis of COPD, chronic bronchitis, or emphysema.

At both baseline and resurvey, participants were repeatedly asked their weight at age 25. The Pearson correlation coefficient of weight at age 25 between two surveys at a mean interval of 7.98 years was 0.77. In the present analysis, we used weight at age 25 self-reported at baseline, but, if available, replacing missing values with weight at age 25 self-reported at resurvey (*n* = 2421).

We calculated the weight change from early to middle or late adulthood, based on self-reported weight at age 25 and measured weights at baseline and resurvey, as the difference of weight between baseline and age 25, between resurvey and baseline, and between resurvey and age 25. Weight change since age 25 was categorized as follows: loss ≥ 5.0 kg, loss 2.5 to 4.9 kg, loss or gain ≤ 2.4 kg, gain 2.5 to 4.9 kg, gain 5.0 to 9.9 kg, gain 10.0 to 14.9 kg, and gain ≥ 15.0 kg. The categories of weight change were defined according to previous studies and data distribution of the present study population, avoiding uneven and small sample size in some categories.

### Ascertainment of hospitalized fracture

The long-term follow-up of cause-specific morbidity and mortality of all CKB participants was achieved by linkage to local disease and death registries and the national health insurance system (with successful linkage for over 97% of surviving participants), supplemented by annual active follow-up. New fracture cases were mainly identified from health insurance claim database, which captures all episodes of hospitalization. However, fractures that did not require inpatient admission were not ascertained in the present study. The admission date for fracture was taken as the diagnosis date. Diseases were coded according to the International Classification of Diseases, 10th Reversion (ICD-10) by trained staff who were blinded to baseline information.

Any fracture cases were defined as fracture at any part of the body coded by S12, S22, S32, S42, S52, S62, S72, T02, T08, T10, T12, and T14.2. M80 was not included in the present analysis because it indicates pathologic fractures caused by some specific disorder that leads to osteoporosis. Fractures least likely due to osteoporosis and most likely because of severe trauma or cancer were excluded [20]. Any fracture cases were further categorized by fracture site into upper limb, lower limb, central body (spine, pelvis, hip, and others), and other fractures. Detailed information on outcome definitions and categories grouped by fracture site are presented in Supplementary Material and Appendix Table 1.

### Measurement of BMD

At the resurvey of 5% surviving participants, we included calcaneus BMD measurement using the quantitative ultrasound method (GE Achilles EXP II) for the first time. BMD was measured as speed of sound (SOS, m/s) and broadband ultrasound attenuation (BUA, dB/MHz). The stiffness index (SI) was further derived from SOS and BUA, calculated using the following formula:

$$ \mathrm{SI}=0.67\times \mathrm{BUA}+0.28\times \mathrm{SOS}-420 $$

The averaged QUS measurements between the left and right calcaneus were used in the present analysis.

### Statistical analysis

The present study included two parts. First, we used baseline and follow-up data of all eligible CKB participants to examine the association between weight change from age 25 to baseline and subsequent risk of hospitalized fracture. We excluded participants who had a clinical diagnosis of fracture in the last 5 years before baseline (*n* = 13,019), cancer (*n* = 2578), or stroke (*n* = 8884) at any time before baseline, as these conditions may be associated with weight loss, malignancy-related fracture, limited mobility, and the possibility of falling. We also excluded participants who were lost to follow-up shortly after baseline (*n* = 1) or those whose information on either weight at age 25 (*n* = 79,928) or baseline weight (*n* = 2) was missing. The final analyses included 411,812 participants.

Second, we used resurvey data to examine the association between weight change from age 25 to resurvey (further divided into two stages: age 25 to baseline, baseline to resurvey) and BMD. Of 25,034 participants who participated in the resurvey, we undertook same exclusion criteria as the first part, including fracture in the last 5 years of resurvey (*n* = 888), cancer (*n* = 340), or stroke (*n* = 970) before baseline or resurvey, missing on BMD measurements (*n* = 360), weight at age 25 (*n* = 1251), or baseline weight (*n* = 1), leaving 21,453 participants for the final analyses.

For the first part of the analyses, participants contributed person-years at risk until the first diagnosis of fracture endpoint, death, loss to follow-up, or December 31, 2016. We used stratified Cox proportional hazard models to estimate the hazard ratios (HRs) and 95% confidence intervals (CI) for the associations of weight change from age 25 to baseline with the risk of hospitalized fracture, with stratification on survey site and age (in 5-year intervals) and attained age as the underlying time scale.

To examine the robustness of the model, we performed several sensitivity analyses: additionally adjusting for intakes of fish oil or cod liver oil, and vitamin supplements, prevalent asthma, chronic kidney disease, rheumatoid arthritis, and psychiatric disorders, and intention to lose weight during the last year of baseline; excluding participants with prevalent diabetes or COPD; excluding participants who had weight change > 2.5 kg during the last year of baseline; excluding participants aged 65 years and above. The main results showed little change (data not shown).

We performed stratified analyses to examine whether the associations between weight loss and each fracture outcome were consistent across baseline characteristics: sex, age, residence, BMI at age 25 and at baseline, tobacco smoking, level of physical activity, intake of supplementary calcium, iron or zinc, intake of dairy products, and menopausal status (only in women). To avoid small cases in some cells, weight change was regrouped as follows: loss ≥ 2.5 kg, loss or gain ≤ 2.4 kg, and gain ≥ 2.5 kg. We performed a likelihood ratio test for the multiplicative interaction, comparing models with and without interaction terms between the stratifying variable and weight change from age 25 to baseline.

For the second part of the analyses, we used linear regression to assess the association of weight change (age 25 to resurvey, age 25 to baseline, and baseline to resurvey) with BMD. The multivariable model included the same set of covariates as the first part of the analysis, with additional adjustment for age and survey site.

All tests for linear trend across categories of weight change were conducted by assigning the median value to each group and treating it as a continuous variable in a separate model. For testing of multiple primary outcomes, a Bonferroni correction was applied to the significance level that divided 0.05 by the number of outcomes examined (0.05/8 = 0.00625 for fracture analysis; 0.05/3 = 0.0167 for BMD analysis). All statistical analyses were performed with Stata version 15.0.

## Results

### Basic characteristics of study participants

Among the eligible 411,812 participants at baseline, the mean age was 50.8 ± 10.4 years; 57.5% were women; and 47.9% resided in urban areas. Their mean BMI was 21.9 ± 2.6 kg/m^2^ at age 25 and 23.8 ± 3.4 kg/m^2^ at baseline. Median weight change from age 25 to baseline was 4.4 kg (interquartile range 11.7 kg), with 20.7% losing weight and 58.5% gaining weight. Participants with a weight loss of ≥ 5.0 kg from age 25 to baseline had the highest BMI at age 25 and the lowest BMI at baseline; they tended to be men and older, reside in rural areas, smoke tobacco, have COPD, and poor self-rated health status (Table 1).

**Table 1:** Baseline characteristics according to weight change from age 25 to baseline among 411,812 participants

	Weight change from age 25 to baseline (kg)							*P*_trend_
	≤ − 5.0	− 4.9–2.5	− 2.4–2.4	2.5–4.9	5.0–9.9	10.0–14.9	≥ 15.0	
Participants (*n* (%))	53,658 (13.0)	31,598 (7.7)	85,757 (20.8)	45,717 (11.1)	84,370 (20.5)	57,934 (14.1)	52,778 (12.8)	
BMI and weight change (mean (SD))								
BMI at age 25* (kg/m^2^)	24.3 (2.6)	22.9 (2.3)	22.1 (2.3)	21.7 (2.3)	21.4 (2.2)	21.1 (2.2)	20.5 (2.3)	< 0.001
BMI at baseline (kg/m^2^)	20.6 (2.4)	21.4 (2.3)	22.1 (2.4)	23.2 (2.3)	24.3 (2.3)	25.8 (2.3)	28.3 (2.9)	< 0.001
Weight change (kg)	− 9.0 (3.8)	− 3.6 (0.7)	0.1 (1.4)	3.7 (0.7)	7.4 (1.4)	12.2 (1.4)	20.4 (5.1)	< 0.001
Demographic and socioeconomic characteristics								
Age (year (SD))	55.6 (10.7)	51.6 (10.6)	49.6 (10.5)	49.0 (10.2)	49.5 (9.9)	50.4 (9.8)	51.4 (9.8)	< 0.001
Women (%)	49.5	54.6	58.8	63.1	61.8	57.7	52.9	< 0.001
Urban area (%)	34.2	37.0	40.6	46.9	51.7	57.7	64.1	< 0.001
Middle school or above (%)	50.0	50.9	53.0	55.1	55.7	56.1	55.6	< 0.001
Married (%)	89.8	90.9	91.1	91.7	92.2	92.5	92.8	< 0.001
Agricultural and industrial worker (%)	62.1	60.2	58.2	55.4	53.2	50.6	47.6	< 0.001
Household income ≥ 10,000 RMB/year (%)	70.9	72.5	74.0	75.8	76.9	77.9	78.6	< 0.001
Lifestyle factors								
Male current smoker^†^ (%)	77.7	73.0	69.2	66.1	63.3	62.1	61.3	< 0.001
Female current smoker^†^ (%)	4.2	2.9	2.4	2.1	2.2	2.3	2.5	<0.001
Male daily alcohol drinker (%)	22.5	23.0	22.6	22.4	20.9	20.2	19.1	< 0.001
Female daily alcohol drinker (%)	1.1	1.0	1.0	0.9	0.9	1.0	0.9	0.023
Physical activity, MET-hour/day	22.3	22.5	22.0	21.5	21.1	20.5	19.6	< 0.001
Dairy product consumption more than once per week (%)	21.4	21.6	22.2	23.1	23.1	22.6	21.6	0.145
Calcium, iron, or zinc supplementation (%)	6.4	6.6	6.7	7.1	7.3	7.8	8.1	< 0.001
Self-reported conditions (%)								
Diabetes	5.9	4.8	4.5	4.7	5.3	6.4	8.1	< 0.001
COPD	9.4	7.5	6.7	6.4	5.9	5.8	5.5	< 0.001
Self-rated poor health (%)	14.1	10.5	8.7	8.0	8.0	8.7	9.8	< 0.001
Male height (m)	1.6	1.6	1.6	1.7	1.7	1.7	1.7	< 0.001
Female height (m)	1.5	1.5	1.5	1.5	1.5	1.6	1.6	< 0.001
Postmenopausal women (%)	51.6	49.8	49.4	48.6	48.4	48.4	48.1	< 0.001

*BMI*, body mass index; *MET*, metabolic equivalent of task; *COPD*, chronic obstructive pulmonary disease. All values are adjusted for age, sex, and survey site, as appropriate, except for BMI and weight change, age, sex, and region

*BMI at age 25 was calculated using self-reported weight at age 25 and measured height at baseline

^†^Participants who had stopped smoking because of illness were included in the current smokers in the present study

The mean age of the 21,453 participants at resurvey was 58.4 ± 10.0 years; 61.3% were women; 43.6% resided in urban areas; and the mean BMI was 24.2 ± 3.5 kg/m^2^. The median interval between baseline and resurvey was 7.98 years (interquartile range 1.16 years); the median weight change during this period was 0.8 kg (interquartile range 5.1 kg). Participants with maximum weight loss being observed had the lowest BMD measures than others and presented similar characteristics as the baseline (Appendix Table 2).

### Weight loss and hospitalized fracture risk

During a median follow-up of 10.1 years (interquartile range 1.96 years; total person-years 4.1 million), we documented 13,065 cases of first diagnosed fracture hospitalizations, including 3411 (26.1%) upper limb fracture, 3640 (27.9%) lower limb fracture, 1973 (15.1%) spine fracture, 229 (1.8%) pelvis fracture, 1222 (9.4%) hip fracture, 1445 (11.1%) other central body fracture, and 2106 (16.1%) other fracture.

After adjustment for potential confounding by sociodemographic and lifestyle factors and weight at age 25, weight change during adulthood was associated with the risk of any fracture and hip fracture (*P*_trend_, 0.003 and < 0.001, respectively). Compared with participants whose weight was stable (± 2.4 kg), the adjusted HRs (95% CIs) for those with weight loss of ≥ 5.0 kg were 1.07 (1.01 to 1.14) for any fracture and 1.39 (1.17 to 1.66) for hip fracture (*P*, 0.017 and < 0.001, respectively; Table 2). Adjustment for weight at baseline instead of weight at age 25 did not substantially alter the association (HRs [95%CI] 1.06 [1.01, 1.13] for any fracture and 1.23 [1.03, 1.46] for hip fracture). Weight change was generally not associated with the risk of upper limb fracture, lower limb fracture, spine fracture, pelvis fracture, other central body fracture, and other fracture (all *P*_trend_ > 0.00625). The associations of weight change with any fracture, hip fracture, and other six groups of fracture were consistent between men and women (all *P*_interaction_ > 0.00625; Appendix Table 3).

**Table 2:** HRs (95% CIs) for association between weight change from age 25 to baseline and risk of fracture among 411,812 participants

		Weight change from age 25 to baseline (kg)							*P*_trend_
		≤ − 5.0	− 4.9–2.5	− 2.4–2.4	2.5–4.9	5.0–9.9	10.0–14.9	≥ 15.0	
Any fracture									
No. of cases	13,065	2357	1182	2768	1366	2426	1596	1370	
Cases/PYs (1/1000)	3.21	4.64	3.80	3.26	2.99	2.88	2.77	2.62	
Model 1		1.10 (1.04, 1.17)	1.03 (0.96, 1.10)	1.00	0.99 (0.92, 1.05)	0.98 (0.92, 1.03)	0.96 (0.90, 1.02)	0.97 (0.91, 1.04)	< 0.001
Model 2		1.07 (1.01, 1.13)	1.01 (0.95, 1.09)	1.00	0.99 (0.93, 1.06)	0.98 (0.93, 1.04)	0.97 (0.91, 1.03)	0.97 (0.90, 1.03)	< 0.001
Model 3		1.07 (1.01, 1.14)	1.02 (0.95, 1.09)	1.00	0.99 (0.93, 1.06)	0.98 (0.93, 1.04)	0.96 (0.91, 1.03)	0.96 (0.90, 1.03)	0.003
Upper limb fracture									
No. of cases	3411	567	294	763	386	636	414	351	
Cases/PYs (1/1000)	0.83	1.10	0.93	0.89	0.84	0.75	0.71	0.67	
Model 1		0.98 (0.87, 1.09)	0.91 (0.80, 1.04)	1.00	1.00 (0.89, 1.13)	0.93 (0.84, 1.03)	0.92 (0.81, 1.04)	0.97 (0.85, 1.10)	0.588
Model 2		0.95 (0.85, 1.06)	0.90 (0.79, 1.03)	1.00	1.01 (0.89, 1.14)	0.94 (0.85, 1.05)	0.93 (0.83, 1.05)	0.99 (0.87, 1.13)	0.745
Model 3		0.92 (0.82, 1.03)	0.89 (0.78, 1.02)	1.00	1.01 (0.90, 1.15)	0.96 (0.86, 1.06)	0.95 (0.84, 1.08)	1.02 (0.89, 1.17)	0.285
Lower limb fracture									
No. of cases	3640	667	352	784	344	660	457	376	
Cases/PYs (1/1000)	0.89	1.29	1.12	0.91	0.75	0.78	0.79	0.71	
Model 1		1.11 (1.00, 1.24)	1.07 (0.94, 1.21)	1.00	0.88 (0.77, 1.00)	0.95 (0.85, 1.05)	0.99 (0.88, 1.11)	0.98 (0.86, 1.11)	0.015
Model 2		1.08 (0.98, 1.21)	1.06 (0.94, 1.20)	1.00	0.88 (0.78, 1.00)	0.95 (0.86, 1.05)	0.98 (0.87, 1.10)	0.95 (0.83, 1.08)	0.018
Model 3		1.10 (0.99, 1.23)	1.07 (0.94, 1.21)	1.00	0.88 (0.77, 1.00)	0.94 (0.85, 1.05)	0.97 (0.86, 1.10)	0.93 (0.82, 1.06)	0.013
Spine fracture									
No. of cases	1973	311	172	369	221	376	282	242	
Cases/PYs (1/1000)	0.48	0.60	0.55	0.43	0.48	0.44	0.48	0.46	
Model 1		0.98 (0.84, 1.15)	1.08 (0.90, 1.29)	1.00	1.18 (1.00, 1.39)	1.07 (0.93, 1.24)	1.16 (0.99, 1.36)	1.15 (0.98, 1.36)	0.029
Model 2		0.95 (0.82, 1.11)	1.06 (0.89, 1.27)	1.00	1.19 (1.00, 1.40)	1.09 (0.94, 1.26)	1.18 (1.01, 1.38)	1.17 (0.99, 1.38)	0.006
Model 3		0.98 (0.84, 1.15)	1.07 (0.89, 1.29)	1.00	1.18 (1.00, 1.39)	1.08 (0.93, 1.25)	1.16 (0.99, 1.36)	1.14 (0.96, 1.35)	0.071
Pelvis fracture									
No. of cases	229	35	34	51	28	47	20	14	
Cases/PYs (1/1000)	0.06	0.07	0.11	0.06	0.06	0.06	0.03	0.03	
Model 1		0.89 (0.57, 1.37)	1.56 (1.01, 2.41)	1.00	1.12 (0.70, 1.77)	1.10 (0.74, 1.64)	0.75 (0.45, 1.26)	0.70 (0.39, 1.28)	0.166
Model 2		0.83 (0.54, 1.30)	1.53 (0.99, 2.36)	1.00	1.12 (0.71, 1.78)	1.10 (0.74, 1.64)	0.75 (0.44, 1.26)	0.67 (0.37, 1.22)	0.215
Model 3		0.90 (0.57, 1.42)	1.57 (1.01, 2.43)	1.00	1.10 (0.70, 1.75)	1.07 (0.72, 1.60)	0.71 (0.42, 1.21)	0.62 (0.34, 1.15)	0.079
Hip fracture									
No. of cases	1222	337	115	248	105	180	115	122	
Cases/PYs (1/1000)	0.30	0.65	0.36	0.29	0.23	0.21	0.20	0.23	
Model 1		1.35 (1.14, 1.59)	1.04 (0.83, 1.29)	1.00	0.85 (0.68, 1.07)	0.76 (0.62, 0.92)	0.67 (0.54, 0.84)	0.74 (0.59, 0.92)	< 0.001
Model 2		1.32 (1.11, 1.56)	1.03 (0.82, 1.29)	1.00	0.84 (0.66, 1.05)	0.73 (0.60, 0.88)	0.62 (0.49, 0.77)	0.64 (0.51, 0.80)	< 0.001
Model 3		1.39 (1.17, 1.66)	1.05 (0.84, 1.32)	1.00	0.83 (0.66, 1.04)	0.71 (0.58, 0.86)	0.59 (0.47, 0.74)	0.60 (0.47, 0.75)	< 0.001
Other central body fracture									
No. of cases	1445	304	150	280	152	263	161	135	
Cases/PYs (1/1000)	0.35	0.59	0.48	0.33	0.33	0.31	0.28	0.26	
Model 1		1.28 (1.08, 1.50)	1.20 (0.98, 1.46)	1.00	1.16 (0.95, 1.41)	1.15 (0.97, 1.36)	1.07 (0.88, 1.30)	1.12 (0.91, 1.38)	0.209
Model 2		1.21 (1.03, 1.43)	1.17 (0.96, 1.43)	1.00	1.18 (0.97, 1.43)	1.18 (1.00, 1.40)	1.12 (0.92, 1.36)	1.18 (0.95, 1.46)	0.969
Model 3		1.16 (0.98, 1.38)	1.15 (0.94, 1.40)	1.00	1.19 (0.97, 1.44)	1.20 (1.01, 1.42)	1.15 (0.94, 1.40)	1.23 (0.99, 1.52)	0.418
Other fracture									
No. of cases	2106	345	160	474	225	436	252	214	
Cases/PYs (1/1000)	0.51	0.67	0.51	0.55	0.49	0.51	0.43	0.40	
Model 1		1.15 (1.00, 1.33)	0.92 (0.77, 1.10)	1.00	0.96 (0.82, 1.12)	1.04 (0.92, 1.19)	0.93 (0.80, 1.09)	0.90 (0.76, 1.05)	0.020
Model 2		1.14 (0.99, 1.31)	0.92 (0.77, 1.10)	1.00	0.96 (0.82, 1.13)	1.05 (0.92, 1.20)	0.94 (0.81, 1.10)	0.89 (0.76, 1.06)	0.037
Model 3		1.14 (0.98, 1.32)	0.92 (0.77, 1.10)	1.00	0.96 (0.82, 1.13)	1.05 (0.92, 1.20)	0.94 (0.80, 1.10)	0.89 (0.76, 1.06)	0.060

*HR*, hazard ratio; *CI*, confidence interval; *PYs*, person-years; *MET*, metabolic equivalent of task. Multivariable models were adjusted for the following: model 1: sex (men or women); model 2: additionally included education (no formal school, primary school, middle school, high school, college, or university or above); household income (< 2500, 2500–4999, 5000–9999, 10,000–19,999, 20,000–34,999, or ≥ 35,000 RMB/year); occupation (agriculture, industrial, administrative or managerial, professional or technical, sales or service, retired, house wife or husband, self-employed, unemployed, or other); marital status (married, widowed, divorced or separated, or never married); alcohol consumption (never or occasional; former; weekly but not daily; daily consuming 1–14, 15–29, 30–59, or ≥ 60 g of pure alcohol); smoking status (never or occasional; former; daily smoking 1–14, 15–24, or ≥ 25 cigarettes or equivalent tobacco; participants who had stopped smoking because of illness were included in the current smokers); physical activity (MET-h/day); intake frequency of dairy products (assigning 0, 0.5, 2, 5, 7 to the frequency categories of never or rarely, 1–3 days/month, 1–3 days/week, 4–6 days/week, 7 days/week, respectively, and treating the variable as continuous variable); intake of supplementary calcium, iron, or zinc (yes or no); height (meters); prevalent diabetes and COPD (presence or absence); and self-rated health (excellent, good, fair, or poor); model 3: additionally included weight at age 25 (kg)

According to stratified analyses, we did not observe any clinically meaningful interaction that met a predetermined statistical significance of *P* < 0.00625 (Appendix Tables 4–11), except that the association of weight loss with the risk of other central body fracture seemed stronger among those aged < 65 years (*P*_interaction_ < 0.001).

### Weight loss and BMD measured at resurvey

Weight change from age 25 to resurvey was positively associated with three BMD measures, especially after further adjusting for weight at age 25 (*P*_trend_ < 0.0167; Table 3). Compared with participants whose weight was stable (± 2.4 kg), those with weight loss of ≥ 5.0 kg had the lowest BMD measures, with β (95% CI) of − 4.52 (− 5.08 to − 3.96) for BUA, − 4.83 (− 6.98 to − 2.67) for SOS, and − 4.36 (− 5.22 to − 3.49) for SI. When analyzed by sex, the associations of weight change with BMD measures seem to be more apparent among women (all *P*_interaction_ < 0.0167; Appendix Table 12). When we further split the weight change from age 25 to resurvey into two stages, the association of BMD measures with adulthood weight change mainly came from the early stage of age 25 to baseline (Table 4).

**Table 3:** Association between weight change from age 25 to resurvey and BMD measures among 21,453 participants

	Weight change from age 25 to resurvey (kg)							*P*_trend_
	≤ − 5.0	− 4.9–2.5	− 2.4–2.4	2.5–4.9	5.0–9.9	10.0–14.9	≥ 15.0	
Participants (*n* (%))	2653 (12.4)	1443 (6.7)	3950 (18.4)	2342 (10.9)	4535 (21.1)	3370 (15.7)	3160 (14.7)	
BUA (dB/MHz)								
Model 1	− 3.54 (− 4.10, − 2.98)	− 1.44 (− 2.11, − 0.77)	0.00	0.77 (0.20, 1.34)	1.82 (1.34, 2.30)	2.16 (1.64, 2.68)	3.18 (2.65, 3.71)	< 0.001
Model 2	− 3.28 (− 3.84, − 2.73)	− 1.38 (− 2.05, − 0.71)	0.00	0.67 (0.11, 1.24)	1.59 (1.11, 2.06)	1.86 (1.34, 2.37)	2.80 (2.26, 3.33)	< 0.001
Model 3	− 4.52 (− 5.08, − 3.96)	− 1.83 (− 2.49, − 1.16)	0.00	1.02 (0.46, 1.59)	2.13 (1.65, 2.60)	2.70 (2.18, 3.22)	4.09 (3.54, 4.63)	< 0.001
SOS (m/s)								
Model 1	− 4.67 (− 6.77, − 2.56)	− 0.83 (− 3.38, 1.71)	0.00	− 0.09 (− 2.25, 2.06)	2.55 (0.74, 4.35)	0.12 (−1.84, 2.07)	0.28 (−1.72, 2.29)	< 0.001
Model 2	− 3.95 (− 6.05, − 1.84)	− 0.71 (− 3.24, 1.83)	0.00	− 0.00 (− 2.15, 2.15)	2.59 (0.79, 4.39)	0.33 (−1.62, 2.29)	1.33 (−0.71, 3.36)	< 0.001
Model 3	− 4.83 (− 6.98, − 2.67)	− 1.02 (− 3.56, 1.52)	0.00	0.25 (− 1.91, 2.40)	2.97 (1.16, 4.78)	0.93 (−1.04, 2.91)	2.24 (0.15, 4.33)	< 0.001
SI								
Model 1	− 3.66 (− 4.51, − 2.81)	− 1.19 (− 2.22, − 0.16)	0.00	0.49 (− 0.38, 1.36)	1.92 (1.19, 2.65)	1.47 (0.69, 2.26)	2.20 (1.39, 3.01)	< 0.001
Model 2	− 3.29 (− 4.13, − 2.44)	− 1.12 (− 2.14, − 0.10)	0.00	0.45 (− 0.42, 1.32)	1.78 (1.05, 2.50)	1.33 (0.54, 2.12)	2.23 (1.41, 3.05)	< 0.001
Model 3	− 4.36 (− 5.22, − 3.49)	− 1.50 (− 2.52, − 0.48)	0.00	0.75 (− 0.11, 1.62)	2.24 (1.51, 2.97)	2.06 (1.27, 2.86)	3.35 (2.51, 4.19)	< 0.001

*BMD*, bone mineral density; *BUA*, broadband ultrasound attenuation; *SOS*, speed of sound; *SI*, stiffness index; *MET*, metabolic equivalent of task. All covariates, except weight at age 25, were measured at resurvey. Multivariable models were adjusted for the following: model 1: age (years), sex (men or women), and ten survey sites; model 2: additionally included education (no formal school, primary school, middle school, high school, college, or university or above); household income (< 2500, 2500–4999, 5000–9999, 10,000–19,999, 20,000–34,999, 35,000–49,999, 50,000–74,999, 75,000–99,999, or ≥ 100,000 RMB/year); occupation (agriculture, industrial, administrative or managerial, professional or technical, sales or service, retired, house wife or husband, self-employed, unemployed, or other); marital status (married, widowed, divorced or separated, or never married); alcohol consumption (never or occasional; former; weekly but not daily; daily consuming 1–14, 15–29, 30–59, or ≥ 60 g of pure alcohol); smoking status (never or occasional; former; daily smoking 1–14, 15–24, or ≥ 25 cigarettes or equivalent tobacco; participants who had stopped smoking because of illness were included in the current smokers); physical activity (MET-h/day); intake frequencies of milk, yoghurt, and other dairy products (assigning 0, 0.5, 2, 5, 7 to the frequency categories of never or rarely, 1–3 days/month, 1–3 days/week, 4–6 days/week, 7 days/week, respectively, and treating the variables as continuous variables); intake of supplementary calcium, iron, or zinc (yes or no); height (meters); prevalent diabetes and COPD (presence or absence); and self-rated health (excellent, good, fair, or poor); model 3: additionally included weight at age 25 (kg)

**Table 4:** Association between different periods of weight change and BMD measures among 21,453 participants

	Weight change (kg)							*P*_trend_
	≤ − 5.0	− 4.9–2.5	− 2.4–2.4	2.5–4.9	5.0–9.9*	10.0–14.9*	≥ 15.0*	
Participants (*n* (%))								
Age 25 → baseline	2682 (12.5)	1780 (8.3)	4557 (21.2)	2496 (11.6)	4389 (20.5)	2941 (13.7)	2608 (12.2)	
Baseline → resurvey	1575 (7.3)	2552 (11.9)	10,308 (48.0)	3909 (18.2)	3109 (14.5)	--	--	
BUA (dB/MHz)								
Age 25 → baseline	− 3.80 (− 4.35, − 3.25)	− 1.32 (− 1.93, − 0.72)	0.00	1.34 (0.80, 1.87)	2.24 (1.78, 2.70)	2.87 (2.35, 3.39)	4.74 (4.17, 5.30)	< 0.001
Baseline → resurvey	− 2.01 (− 2.61, − 1.41)	− 0.62 (− 1.10, − 0.14)	0.00	0.84 (0.43, 1.24)	0.72 (0.27, 1.17)	--	--	< 0.001
SOS (m/s)								
Age 25 → baseline	− 6.19 (− 8.29, − 4.08)	− 2.36 (− 4.67, − 0.05)	0.00	1.26 (− 0.79, 3.31)	3.11 (1.35, 4.87)	2.28 (0.28, 4.28)	3.82 (1.65, 5.98)	< 0.001
Baseline → resurvey	1.26 (− 1.04, 3.56)	− 0.05 (− 1.89, 1.78)	0.00	0.26 (− 1.30, 1.82)	− 2.63 (− 4.35, − 0.91)	--	--	0.010
SI								
Age 25 → baseline	− 4.25 (− 5.10, − 3.41)	− 1.54 (− 2.46, − 0.61)	0.00	1.24 (0.42, 2.07)	2.35 (1.65, 3.06)	2.55 (1.74, 3.35)	4.22 (3.35, 5.09)	< 0.001
Baseline → resurvey	− 0.99 (− 1.92, − 0.07)	− 0.43 (− 1.16, 0.31)	0.00	0.63 (0.00, 1.26)	− 0.25 (− 0.94, 0.44)	--	--	0.056

*BMD*, bone mineral density; *BUA*, broadband ultrasound attenuation; *SOS*, speed of sound; *SI*, stiffness index. All covariates, except weight at age 25 and at baseline, were measured at resurvey. Multivariable models were adjusted for the same set of covariates as in the model 3 of Table 3, except for replacing the weight at age 25 with the weight at the beginning of the corresponding period (kg)

*Combined these three categories of weight gain into one category when analyzing the association of weight change from baseline to resurvey with BMD measures

## Discussion

In this large, prospective cohort study of middle-aged and older Chinese adults, we found that participants who lost weight ≥ 5 kg from young adulthood through the middle ages had a 39% higher risk of hip fracture in the subsequent 10 years than those with stable weight. There was no difference in the risk of fracture at other sites between weight change groups. The participants with the long-term weight loss also showed lower BMD measures, to which the weight loss during the early stage of adulthood contributed most.

The Iowa Women’s Health Study of 26,814 women aged 55–69 years showed that maintained weight loss between 18 years and baseline age was associated with an increased risk of hip fracture (RR = 1.99; 95% CI 1.06, 3.74) but not with total fractures risk (RR = 1.05; 95% CI 0.87, 1.27) in 6 years, using the combined categories of stable weight and small gain as the reference group [12]. In this study, maintained weight loss was defined as those who lost weight > 10% during any 10-year interval and subsequently maintained weight within ± 5% of the reduced value. The results of our study further consolidate these findings and add to evidence that the risk of other site fractures was not affected by weight loss between early and middle adulthood.

In the Longitudinal Aging Study Amsterdam of 264 men and 258 women aged 65 years and older, body weight increase since the age 25 was positively associated with BMD in women [14]. However, the result of the weight loss participants was not presented separately. In another study of 749 Japanese women aged 40–74 years, women who were normal or overweight at 20 years but underweight at baseline had higher odds of having osteopenia than those with normal or overweight at both age 20 and baseline (OR = 2.95; 95% CI 1.67–5.24) [21]. The findings in our study are consistent with these previous reports and indicate that the more the weight loss since young adulthood, the lower BMD the participants would have in their middle or late adulthood. A similar loss of weight that occurred from age 25 to their middle age had a more negative impact on BMD than that occurred in the decade of the middle or late adulthood.

In the present Chinese population, the results, based on both the hospitalized fracture risk and BMD, were consistent with the hypothesis that the long-term weight loss since young adulthood harms bone health later in life. Such an association was not attenuated with adjustment for the weight at age 25, suggesting an influence that is independent of weight and potential peak bone mass attained in young adults. Also, most of the previous studies focused on the weight loss that occurs during middle and late adulthood and had linked it to lower BMD [8–10] and increased risks of hip fracture [4–7] and fracture at other sites, such as spine [7], clavicle [7], upper limb [6], and distal forearm [22]. All these findings together suggest that weight loss at any stage of adulthood may associate with increased bone loss and fracture risk, particularly the risk of hip fracture. Potential mechanisms underlying this association include inadequate intakes of dietary calcium [23], vitamin D [24], and protein [25], and changes in mechanical loading [26], muscle mass [27], fat mass [14], and hormonal regulation on bone metabolism, such as estrogen [28], adiponectin [29], leptin [30], sex hormone-binding globulin (SHBG) [31, 32].

The present study of the Chinese population comprehensively investigated the associations of weight loss since young adulthood with both the fracture risk at various sites and the BMD in later life. The hospitalized fractures were mainly identified through linkage to health insurance claim database. The fracture incidence in our population was comparable with a self-reported traumatic fracture incidence collected from a nationally representative sample of 512,187 participants from eight provinces of China in 2014 [33]. The strengths of the study also included measured weight at both baseline and resurvey, a large study population of both men and women, the inclusion of the Chinese population living in urban and rural areas with different socioeconomic status, and careful adjustment for potential confounders.

Some limitations of the present study are acknowledged. First, underestimation of fracture incidence was inevitable since minor fractures that do not result in hospitalization were not captured in the present study. Second, BMD was measured by calcaneal QUS, a radiation-free, portable, and low-cost method commonly used in large-scale population study [34]. However, QUS measures are correlated with BMD measured by the standard method of dual-energy X-ray absorptiometry [35, 36]. Third, BMD was measured only once at resurvey that prevented us from knowing the actual change in BMD during adulthood. Fourth, the timing of weight change between age 25 and baseline was uncertain due to the lack of repeated measures of body weight. Also, by comparing the associations of fracture risk following the resurvey with weight change between age 25 and baseline to that between baseline and resurvey, we may address the question of which of the stage during which weight loss occurs contributed most to the fracture risk in later life. However, during a median follow-up of 2.9 years after resurvey until the end of 2016, small fracture cases resulted in insufficient statistical power to answer the question. With an extended follow-up of the CKB study, future analysis will be able to examine whether weight loss during early and middle adulthood or weight loss during old age is more important in influencing hip fracture risk. Also, we have no information about the reasons for the long-term weight loss. Fifth, we were unable to examine the biological mechanism underlying the association between weight loss and increased risk of hip fracture due to the lack of measures of lower extremity physical performance, relevant biomarkers, and fall history. Finally, although we adjusted for potential confounders such as socioeconomic status, lifestyle, and comorbidity, residual confounding by other unmeasured or unknown factors may still exist.

Findings of this large prospective study of the Chinese population indicate that a weight loss of ≥ 5 kg from early adulthood to midlife was associated with a higher risk of hip fracture and lower BMD in later life. If losing weight is necessary for greater health benefits for people with overweight or obesity or is insisted for any other reasons, the risks of weight loss on later life bone health and any measures that may prevent or delay rapid bone loss should be advised.

## Electronic Supplementary Materials

ESM 1 (DOCX 144 kb)
