# Supplementary material for: Weight loss since early adulthood, later life risk of fracture hospitalizations, and bone mineral density: a prospective cohort study of 0.5 million Chinese adults
Source: Arch Osteoporos. 2020 Apr 19;15(1):60. doi: 10.1007/s11657-020-00734-3 (PMC7167376; doi:10.1007/s11657-020-00734-3)
Supplement: Supplementary file 1 — (DOCX 144 kb) [file 11657_2020_734_MOESM1_ESM.docx]

**Supplementary Appendix**

**Table of contents**

[**Members of the China Kadoorie Biobank collaborative group** 2](#_Toc34907488)

[**Outcome definitions for any fracture** 4](#_Toc34907489)

[Appendix table 1. ICD10 codes and classification of fracture. 6](#_Toc34907490)

[Appendix table 2. Resurvey characteristics according to weight change from age 25 to resurvey among 21,453 participants. 9](#_Toc34907491)

[Appendix table 3. HRs (95% CIs) for association between weight change from age 25 to baseline and risk of fracture among 175,157 men and 236,655 women. 12](#_Toc34907492)

[Appendix table 4. Association between weight change from age 25 to baseline and risk of any fracture according to baseline factors. 16](#_Toc34907493)

[Appendix table 5. Association between weight change from age 25 to baseline and risk of upper limb fracture according to baseline factors. 18](#_Toc34907494)

[Appendix table 6. Association between weight change from age 25 to baseline and risk of lower limb fracture according to baseline factors. 20](#_Toc34907495)

[Appendix table 7. Association between weight change from age 25 to baseline and risk of spine fracture according to baseline factors. 22](#_Toc34907496)

[Appendix table 8. Association between weight change from age 25 to baseline and risk of pelvis fracture according to baseline factors. 24](#_Toc34907497)

[Appendix table 9. Association between weight change from age 25 to baseline and risk of hip fracture according to baseline factors. 26](#_Toc34907498)

[Appendix table 10. Association between weight change from age 25 to baseline and risk of other central body fracture according to baseline factors. 28](#_Toc34907499)

[Appendix table 11. Association between weight change from age 25 to baseline and risk of other fracture according to baseline factors. 30](#_Toc34907500)

[Appendix table 12. Association between weight change from age 25 to resurvey and BMD measures among 8,295 men and 13,158 women. 32](#_Toc34907501)

**Members of the China Kadoorie Biobank collaborative group**

**International Steering Committee:** Junshi Chen, Zhengming Chen (PI), Robert Clarke, Rory Collins, Yu Guo, Liming Li (PI), Jun Lv, Richard Peto, Robin Walters. **International Co-ordinating Centre, Oxford:** Daniel Avery, Ruth Boxall, Derrick Bennett, Yumei Chang, Yiping Chen, Zhengming Chen, Robert Clarke, Huaidong Du, Simon Gilbert, Alex Hacker, Mike Hill, Michael Holmes, Andri Iona, Christiana Kartsonaki, Rene Kerosi, Ling Kong, Om Kurmi, Garry Lancaster, Sarah Lewington, Kuang Lin, John McDonnell, Iona Millwood, Qunhua Nie, Jayakrishnan Radhakrishnan, Paul Ryder, Sam Sansome, Dan Schmidt, Paul Sherliker, Rajani Sohoni, Becky Stevens, Iain Turnbull, Robin Walters, Jenny Wang, Lin Wang, Neil Wright, Ling Yang, Xiaoming Yang. **National Co-ordinating Centre, Beijing:** Zheng Bian, Yu Guo, Xiao Han, Can Hou, Jun Lv, Pei Pei, Chao Liu, Yunlong Tan, Canqing Yu. **10 Regional Co-ordinating Centres: Qingdao CDC:** Zengchang Pang, Ruqin Gao, Shanpeng Li, Shaojie Wang, Yongmei Liu, Ranran Du, Yajing Zang, Liang Cheng, Xiaocao Tian, Hua Zhang, Yaoming Zhai, Feng Ning, Xiaohui Sun, Feifei Li. **Licang CDC:** Silu Lv, Junzheng Wang, Wei Hou. **Heilongjiang Provincial CDC:** Mingyuan Zeng, Ge Jiang, Xue Zhou. **Nangang CDC:** Liqiu Yang, Hui He, Bo Yu, Yanjie Li, Qinai Xu,Quan Kang, Ziyan Guo. **Hainan Provincial CDC:** Dan Wang, Ximin Hu, Jinyan Chen, Yan Fu, Zhenwang Fu, Xiaohuan Wang. **Meilan CDC:** Min Weng, Zhendong Guo, Shukuan Wu,Yilei Li, Huimei Li, Zhifang Fu. **Jiangsu Provincial CDC:** Ming Wu, Yonglin Zhou, Jinyi Zhou, Ran Tao, Jie Yang, Jian Su. **Suzhou CDC:** Fang liu, Jun Zhang, Yihe Hu, Yan Lu, , Liangcai Ma, Aiyu Tang, Shuo Zhang, Jianrong Jin, Jingchao Liu. **Guangxi Provincial CDC:** Zhenzhu Tang, Naying Chen, Ying Huang. **Liuzhou CDC:** Mingqiang Li, Jinhuai Meng, Rong Pan, Qilian Jiang, Jian Lan,Yun Liu, Liuping Wei, Liyuan Zhou, Ningyu Chen Ping Wang, Fanwen Meng, Yulu Qin,, Sisi Wang. **Sichuan Provincial CDC:** Xianping Wu, Ningmei Zhang, Xiaofang Chen,Weiwei Zhou. **Pengzhou CDC:** Guojin Luo, Jianguo Li, Xiaofang Chen, Xunfu Zhong, Jiaqiu Liu, Qiang Sun. **Gansu Provincial CDC:** Pengfei Ge, Xiaolan Ren, Caixia Dong. **Maiji CDC:** Hui Zhang, Enke Mao, Xiaoping Wang, Tao Wang, Xi zhang. **Henan Provincial CDC:** Ding Zhang, Gang Zhou, Shixian Feng, Liang Chang, Lei Fan. **Huixian CDC:** Yulian Gao, Tianyou He, Huarong Sun, Pan He, Chen Hu, Xukui Zhang, Huifang Wu, Pan He. **Zhejiang Provincial CDC:** Min Yu, Ruying Hu, Hao Wang. Tongxiang CDC: Yijian Qian, Chunmei Wang, Kaixu Xie, Lingli Chen, Yidan Zhang, Dongxia Pan, Qijun Gu. **Hunan Provincial CDC:** Yuelong Huang, Biyun Chen, Li Yin, Huilin Liu, Zhongxi Fu, Qiaohua Xu. **Liuyang CDC:** Xin Xu, Hao Zhang, Huajun Long, Xianzhi Li, Libo Zhang, Zhe Qiu.

**Outcome definitions for any fracture**

Any fracture cases were defined as fracture at any part of the body coded by S12, S22, S32, S42, S52, S62, S72, T02, T08, T10, T12, and T14.2. The S section provides codes for the fractures related to single body regions; the T section covers fractures at multiple or unspecified body regions. M80 was not included in the present analysis because it indicates pathologic fractures caused by some specific disorder that leads to osteoporosis.

A multidisciplinary expert panel has developed osteoporosis attribution scores for all fracture sites according to their likelihood of being due to osteoporosis^1^. A score of 1 indicates a fracture least likely to be osteoporosis-related and a score of 9 indicates a fracture most likely to be osteoporosis-related. The panel concluded that fractures receiving the lowest attribution scores (1-3) were least likely due to osteoporosis and most likely because of severe trauma or cancer-related fractures, including open fractures of the proximal humerus and closed fractures of the skull, face, toe, scapula, and finger; atypical fractures (i.e. flail chest, larynx and trachea); and multiple fractures involving both lower limb(s), lower with upper limb and lower limb(s) with rib(s) and sternum. Accordingly, we excluded all fractures scored 1-3 in all clinical fracture risk groups from our analysis, with some exceptions. Open fractures of the proximal humerus were not excluded because health insurance claim database does not distinguish between open and closed fractures. The ICD-10 guidelines note that a fracture not indicated as closed or open should be classified as closed. Multiple fractures involving lower limb(s) with rib(s) was not excluded because there was no corresponding ICD-10 code. Detailed information on the inclusion and exclusion criteria and categories grouped by fracture site are presented in appendix table 1.

**References**

1. Warriner AH, Patkar NM, Curtis JR, et al. Which fractures are most attributable to osteoporosis? *J Clin Epidemiol* 2011;64(1):46-53. doi: 10.1016/j.jclinepi.2010.07.007

Appendix table 1. ICD10 codes and classification of fracture.

| ICD-10 codes | ICD-10 definition | Categories grouped by fracture site |
| --- | --- | --- |
| S02 | Fracture of skull and facial bones | Excluded |
| S12.0 | Fracture of first cervical vertebra | Spine |
| S12.1 | Fracture of second cervical vertebra | Spine |
| S12.2 | Fracture of other specified cervical vertebra | Spine |
| S12.7 | Multiple fractures of cervical spine | Spine |
| S12.8 | Fracture of other parts of neck (hyoid bone, larynx, thyroid cartilage, trachea) | Excluded |
| S12.9 | Fracture of neck, part unspecified | Other central body |
| S22.0 | Fracture of thoracic vertebra | Spine |
| S22.1 | Multiple fracture of thoracic spine | Spine |
| S22.2 | Fracture of sternum | Other central body |
| S22.3 | Fracture of rib | Other central body |
| S22.4 | Multiple fractures of ribs | Other central body |
| S22.5 | Flail chest | Excluded |
| S22.8 | Fracture of other parts of bony thorax | Other central body |
| S22.9 | Fracture of bony thorax, part unspecified | Other central body |
| S32.0 | Fracture of lumbar vertebra | Spine |
| S32.1 | Fracture of sacrum | Pelvis |
| S32.2 | Fracture of coccyx | Pelvis |
| S32.3 | Fracture of ilium | Pelvis |
| S32.4 | Fracture of acetabulum | Pelvis |
| S32.5 | Fracture of pubis | Pelvis |
| S32.7 | Multiple fractures of lumbar spine and pelvis | Other central body |
| S32.8 | Fracture of other and unspecified parts of lumbar spine and pelvis | Other central body |
| S42.0 | Fracture of clavicle | Upper limb |
| S42.1 | Fracture of scapula | Excluded |
| S42.2 | Fracture of upper end of humerus | Upper limb |
| S42.3 | Fracture of shaft of humerus | Upper limb |
| S42.4 | Fracture of lower end of humerus | Upper limb |
| S42.7 | Multiple fractures of clavicle, scapula and humerus | Upper limb |
| S42.8 | Fracture of other parts of shoulder and upper arm | Upper limb |
| S42.9 | Fracture of shoulder girdle, part unspecified | Upper limb |
| S52.0 | Fracture of upper end of ulna | Upper limb |
| S52.1 | Fracture of upper end of radius | Upper limb |
| S52.2 | Fracture of shaft of ulna | Upper limb |
| S52.3 | Fracture of shaft of radius | Upper limb |
| S52.4 | Fracture of shafts of both ulna and radius | Upper limb |
| S52.5 | Fracture of lower end of radius | Upper limb |
| S52.6 | Fracture of lower end of both ulna and radius | Upper limb |
| S52.7 | Multiple fractures of forearm | Upper limb |
| S52.8 | Fracture of other parts of forearm | Upper limb |
| S52.9 | Fracture of forearm, part unspecified | Upper limb |
| S62.0 | Fracture of navicular [scaphoid] bone of hand | Upper limb |
| S62.1 | Fracture of other carpal bone(s) | Upper limb |
| S62.2 | Fracture of first metacarpal bone | Upper limb |
| S62.3 | Fracture of other metacarpal bone | Upper limb |
| S62.4 | Multiple fractures of metacarpal bones | Upper limb |
| S62.5 | Fracture of thumb | Excluded |
| S62.6 | Fracture of other finger | Excluded |
| S62.7 | Multiple fractures of fingers | Excluded |
| S62.8 | Fracture of other and unspecified parts of wrist and hand | Upper limb |
| S72.0 | Fracture of neck of femur | Hip |
| S72.1 | Pertrochanteric fracture | Hip |
| S72.2 | Subtrochanteric fracture | Hip |
| S72.3 | Fracture of shaft of femur | Lower limb |
| S72.4 | Fracture of lower end of femur | Lower limb |
| S72.7 | Multiple fractures of femur | Lower limb |
| S72.8 | Fracture of other parts of femur | Lower limb |
| S72.9 | Fracture of femur, part unspecified | Lower limb |
| S82.0 | Fracture of patella | Lower limb |
| S82.1 | Fracture of upper end of tibia | Lower limb |
| S82.2 | Fracture of shaft of tibia | Lower limb |
| S82.3 | Fracture of lower end of tibia | Lower limb |
| S82.4 | Fracture of fibula alone | Lower limb |
| S82.5 | Fracture of medical malleolus | Lower limb |
| S82.6 | Fracture of lateral malleolus | Lower limb |
| S82.7 | Multiple fractures of lower leg | Lower limb |
| S82.8 | Fracture of other parts of lower leg | Lower limb |
| S82.9 | Fracture of lower leg, part unspecified | Lower limb |
| S92.0 | Fracture of calcaneus | Lower limb |
| S92.1 | Fracture of talus | Lower limb |
| S92.2 | Fracture of other tarsal bone(s) | Lower limb |
| S92.3 | Fracture of metatarsal bone | Lower limb |
| S92.4 | Fracture of great toe | Excluded |
| S92.5 | Fracture of other toe | Excluded |
| S92.7 | Multiple fractures of foot | Lower limb |
| S92.9 | Fracture of foot, unspecified | Lower limb |
| T02.0 | Fractures involving head with neck | Other |
| T02.1 | Fractures involving thorax with lower back and pelvis | Other central body |
| T02.2 | Fractures involving multiple regions of one upper limb | Upper limb |
| T02.3 | Fractures involving multiple regions of one lower limb | Lower limb |
| T02.4 | Fractures involving multiple regions of both upper limbs | Upper limb |
| T02.5 | Fractures involving multiple regions of both lower limbs | Excluded |
| T02.6 | Fractures involving multiple regions of upper limb(s) with lower limb(s) | Excluded |
| T02.7 | Fractures involving thorax with lower back and pelvis with limb(s) | Other |
| T02.8 | Fractures involving other combinations of body regions | Other |
| T02.9 | Multiple fractures, unspecified | Other |
| T08 | Fracture of spine, level unspecified | Spine |
| T10 | Fracture of upper limb, level unspecified | Upper limb |
| T12 | Fracture of upper limb, level un | Lower limb |
| T14.2 | Fracture of unspecified body region | Other |

Appendix table 2. Resurvey characteristics according to weight change from age 25 to resurvey among 21,453 participants.

|  | Weight change from age 25 to resurvey, kg | | | | | | | P_trend_ |
| --- | --- | --- | --- | --- | --- | --- | --- | --- |
|  | ≤-5.0 | -4.9--2.5 | -2.4-2.4 | 2.5-4.9 | 5.0-9.9 | 10.0-14.9 | ≥15.0 |  |
| Participants, n (%) | 2,653 (12.4) | 1,443 (6.7) | 3,950 (18.4) | 2,342 (10.9) | 4,535 (21.1) | 3,370 (15.7) | 3,160 (14.7) |  |
| BMI and weight change, mean (SD) |  |  |  |  |  |  |  |  |
| BMI at age 25^*^, kg/m^2^ | 24.2 (2.7) | 23.0 (2.4) | 22.3 (2.4) | 21.8 (2.3) | 21.5 (2.2) | 21.1 (2.2) | 20.5 (2.4) | <0.001 |
| BMI at baseline, kg/m^2^ | 21.2 (2.7) | 21.8 (2.6) | 22.4 (2.7) | 23.0 (2.6) | 23.9 (2.7) | 25.2 (2.7) | 27.3 (3.2) | <0.001 |
| BMI at resurvey, kg/m^2^ | 20.5 (2.5) | 21.7 (2.4) | 22.5 (2.4) | 23.4 (2.3) | 24.6 (2.3) | 26.0 (2.4) | 28.5 (3.0) | <0.001 |
| Weight change from age 25 to baseline, kg | -7.4 (4.8) | -3.0 (3.7) | 0.1 (3.8) | 2.9 (3.8) | 6.1 (4.1) | 10.3 (4.3) | 17.8 (6.6) | <0.001 |
| Weight change from baseline to resurvey, kg | -2.1 (4.1) | -0.6 (3.6) | 0.1 (3.6) | 0.8 (3.7) | 1.4 (3.9) | 1.9 (4.2) | 2.8 (4.8) | <0.001 |
| Weight change from age 25 to resurvey, kg | -9.5 (4.0) | -3.6 (0.7) | 0.1 (1.4) | 3.7 (0.7) | 7.4 (1.4) | 12.3 (1.4) | 20.6 (5.3) | <0.001 |
| BMD measures, mean (SD) |  |  |  |  |  |  |  |  |
| BUA, dB/MHz | 104.1 (13.1) | 107.3 (12.6) | 109.2 (12.5) | 110.3 (12.3) | 111.5 (11.9) | 112.2 (11.6) | 113.7 (11.2) | <0.001 |
| SOS, m/s | 1544.7 (47.0) | 1552.7 (36.1) | 1556.2 (47.5) | 1558.1 (56.4) | 1560.4 (43.1) | 1558.7 (44.8) | 1559.0 (36.0) | <0.001 |
| SI | 81.8 (19.6) | 86.2 (16.4) | 88.4 (19.1) | 89.7 (21.2) | 91.1 (17.7) | 91.1 (18.0) | 92.2 (15.5) | <0.001 |
| Demographic and socioeconomic characteristics |  |  |  |  |  |  |  |  |
| Age, year | 64.1 (9.8) | 60.2 (10.1) | 58.3 (10.1) | 57.0 (9.9) | 56.9 (9.5) | 57.0 (9.5) | 57.3 (9.6) | <0.001 |
| Women, % | 56.0 | 60.3 | 63.4 | 65.1 | 65.2 | 61.4 | 55.2 | 0.167 |
| Urban area, % | 32.5 | 33.9 | 38.6 | 43.3 | 44.9 | 49.8 | 55.1 | <0.001 |
| Middle school or above, % | 45.9 | 46.8 | 48.9 | 49.8 | 50.2 | 49.8 | 50.8 | <0.001 |
| Married, % | 87.2 | 86.5 | 88.2 | 87.6 | 88.9 | 88.6 | 89.0 | 0.003 |
| Agricultural and industrial worker, % | 43.4 | 43.0 | 39.8 | 39.7 | 38.6 | 37.9 | 33.8 | <0.001 |
| Household income ≥50 000 RMB/year, % | 41.9 | 40.9 | 43.1 | 45.8 | 45.5 | 45.1 | 45.4 | <0.001 |
| Lifestyle factors |  |  |  |  |  |  |  |  |
| Male current smoker^†^, % | 68.2 | 63.5 | 59.0 | 58.9 | 56.0 | 53.7 | 52.8 | <0.001 |
| Female current smoker^†^, % | 3.4 | 2.7 | 1.8 | 1.7 | 1.8 | 1.6 | 1.9 | 0.001 |
| Male daily alcohol drinker, % | 21.5 | 22.8 | 20.8 | 22.1 | 19.9 | 20.1 | 19.5 | 0.085 |
| Female daily alcohol drinker, % | 1.9 | 1.3 | 1.2 | 1.4 | 1.5 | 1.0 | 1.3 | 0.204 |
| Physical activity, MET-hour/day | 18.9 | 19.7 | 19.3 | 19.1 | 18.8 | 18.2 | 17.3 | <0.001 |
| Dairy product consumption more than once per week, % |  |  |  |  |  |  |  |  |
| Yoghurt | 11.6 | 11.4 | 11.8 | 10.9 | 10.8 | 11.1 | 11.2 | 0.434 |
| Milk | 20.6 | 19.5 | 20.4 | 20.8 | 19.5 | 19.1 | 19.4 | 0.096 |
| Other dairy products | 5.7 | 6.1 | 4.3 | 5.5 | 4.8 | 4.6 | 4.3 | 0.012 |
| Calcium, iron, or zinc supplementation, % | 18.9 | 18.6 | 17.4 | 16.7 | 17.9 | 20.4 | 19.1 | 0.122 |
| Self-reported conditions, % |  |  |  |  |  |  |  |  |
| Diabetes | 6.7 | 6.5 | 7.3 | 8.1 | 8.8 | 11.1 | 14.7 | <0.001 |
| COPD | 32.6 | 28.0 | 26.7 | 26.5 | 24.7 | 22.9 | 23.3 | <0.001 |
| Self-rated poor health | 14.9 | 11.6 | 11.0 | 10.0 | 10.9 | 10.1 | 12.2 | 0.002 |
| Male height m | 1.6 | 1.6 | 1.6 | 1.6 | 1.6 | 1.7 | 1.7 | <0.001 |
| Female height, m | 1.5 | 1.5 | 1.5 | 1.5 | 1.5 | 1.5 | 1.6 | <0.001 |
| Postmenopausal women, % | 76.0 | 74.8 | 75.4 | 73.8 | 74.9 | 73.8 | 73.8 | 0.019 |

BMI, body mass index; BMD, bone mineral density; BUA, broadband ultrasound attenuation; SOS, speed of sound; SI, stiffness index; MET, metabolic equivalent of task; COPD, chronic obstructive pulmonary disease.

All values are adjusted for age, sex, and survey site, as appropriate, except for BMI and weight change, BMD measures, age, sex, and region.

^*^BMI at age 25 was calculated using self-reported weight at age 25 and measured height at baseline

^†^Participants who had stopped smoking because of illness were included in the current smokers in the present study.

Appendix table 3. HRs (95% CIs) for association between weight change from age 25 to baseline and risk of fracture among 175,157 men and 236,655 women.

|  |  | Weight change from age 25 to baseline, kg | | | | | | | P_trend_ | P_interaction_ |
| --- | --- | --- | --- | --- | --- | --- | --- | --- | --- | --- |
|  |  | ≤-5.0 | -4.9--2.5 | -2.4-2.4 | 2.5-4.9 | 5.0-9.9 | 10.0-14.9 | ≥15.0 |  |  |
| Any fracture |  |  |  |  |  |  |  |  |  | 0.041 |
| Men |  |  |  |  |  |  |  |  |  |  |
| No. of cases | 5,008 | 1,040 | 519 | 1,113 | 467 | 824 | 551 | 494 |  |  |
| Cases/PYs (1/1000) | 2.94 | 4.16 | 3.75 | 3.24 | 2.82 | 2.60 | 2.27 | 2.01 |  |  |
| HR (95% CI) |  | 1.10 (1.01, 1.21) | 1.05 (0.94, 1.16) | 1.00 | 0.94 (0.85, 1.05) | 0.93 (0.84, 1.01) | 0.89 (0.80, 0.99) | 0.89 (0.79, 0.99) | <0.001 |  |
| Women |  |  |  |  |  |  |  |  |  |  |
| No. of cases | 8,057 | 1,317 | 663 | 1,655 | 899 | 1,602 | 1,045 | 876 |  |  |
| Cases/PYs (1/1000) | 3.41 | 5.10 | 3.85 | 3.27 | 3.09 | 3.06 | 3.12 | 3.15 |  |  |
| HR (95% CI) |  | 1.06 (0.98, 1.14) | 0.99 (0.90, 1.08) | 1.00 | 1.03 (0.95, 1.12) | 1.01 (0.95, 1.09) | 0.99 (0.91, 1.07) | 0.98 (0.90, 1.07) | 0.232 |  |
| Upper limb fracture |  |  |  |  |  |  |  |  |  | 0.119 |
| Men |  |  |  |  |  |  |  |  |  |  |
| No. of cases | 1,006 | 172 | 106 | 235 | 115 | 156 | 122 | 100 |  |  |
| Cases/PYs (1/1000) | 0.58 | 0.68 | 0.76 | 0.68 | 0.69 | 0.49 | 0.50 | 0.40 |  |  |
| HR (95% CI) |  | 0.88 (0.71, 1.09) | 0.99 (0.78, 1.25) | 1.00 | 1.10 (0.88, 1.37) | 0.82 (0.67, 1.01) | 0.93 (0.74, 1.16) | 0.89 (0.69, 1.13) | 0.563 |  |
| Women |  |  |  |  |  |  |  |  |  |  |
| No. of cases | 2,405 | 395 | 188 | 528 | 271 | 480 | 292 | 251 |  |  |
| Cases/PYs (1/1000) | 1.01 | 1.51 | 1.08 | 1.03 | 0.93 | 0.91 | 0.87 | 0.90 |  |  |
| HR (95% CI) |  | 0.94 (0.82, 1.08) | 0.83 (0.70, 0.98) | 1.00 | 0.99 (0.85, 1.15) | 0.99 (0.88, 1.13) | 0.92 (0.80, 1.07) | 1.01 (0.86, 1.19) | 0.414 |  |
| Lower limb fracture |  |  |  |  |  |  |  |  |  | 0.138 |
| Men |  |  |  |  |  |  |  |  |  |  |
| No. of cases | 1,496 | 311 | 163 | 337 | 134 | 236 | 169 | 146 |  |  |
| Cases/PYs (1/1000) | 0.87 | 1.23 | 1.17 | 0.97 | 0.80 | 0.74 | 0.69 | 0.59 |  |  |
| HR (95% CI) |  | 1.10 (0.94, 1.30) | 1.06 (0.88, 1.28) | 1.00 | 0.88 (0.72, 1.07) | 0.84 (0.71, 1.00) | 0.85 (0.70, 1.02) | 0.80 (0.65, 0.98) | <0.001 |  |
| Women |  |  |  |  |  |  |  |  |  |  |
| No. of cases | 2,144 | 356 | 189 | 447 | 210 | 424 | 288 | 230 |  |  |
| Cases/PYs (1/1000) | 0.90 | 1.36 | 1.08 | 0.88 | 0.72 | 0.80 | 0.85 | 0.82 |  |  |
| HR (95% CI) |  | 1.10 (0.95, 1.28) | 1.06 (0.89, 1.26) | 1.00 | 0.88 (0.75, 1.04) | 1.01 (0.88, 1.15) | 1.04 (0.90, 1.21) | 1.01 (0.85, 1.19) | 0.499 |  |
| Spine fracture |  |  |  |  |  |  |  |  |  | 0.177 |
| Men |  |  |  |  |  |  |  |  |  |  |
| No. of cases | 533 | 111 | 59 | 102 | 43 | 92 | 62 | 64 |  |  |
| Cases/PYs (1/1000) | 0.31 | 0.44 | 0.42 | 0.29 | 0.26 | 0.29 | 0.25 | 0.26 |  |  |
| HR (95% CI) |  | 1.18 (0.89, 1.56) | 1.24 (0.90, 1.72) | 1.00 | 0.95 (0.66, 1.35) | 1.11 (0.84, 1.48) | 1.07 (0.77, 1.48) | 1.21 (0.86, 1.69) | 0.982 |  |
| Women |  |  |  |  |  |  |  |  |  |  |
| No. of cases | 1,440 | 200 | 113 | 267 | 178 | 284 | 220 | 178 |  |  |
| Cases/PYs (1/1000) | 0.60 | 0.76 | 0.65 | 0.52 | 0.61 | 0.54 | 0.65 | 0.63 |  |  |
| HR (95% CI) |  | 0.90 (0.74, 1.09) | 1.00 (0.80, 1.25) | 1.00 | 1.26 (1.05, 1.53) | 1.07 (0.90, 1.26) | 1.18 (0.98, 1.42) | 1.09 (0.89, 1.34) | 0.047 |  |
| Pelvis fracture |  |  |  |  |  |  |  |  |  | 0.782 |
| Men |  |  |  |  |  |  |  |  |  |  |
| No. of cases | 71 | 16 | 13 | 13 | 9 | 11 | 6 | 3 |  |  |
| Cases/PYs (1/1000) | 0.04 | 0.06 | 0.09 | 0.04 | 0.05 | 0.03 | 0.02 | 0.01 |  |  |
| HR (95% CI) |  | 1.42 (0.65, 3.09) | 2.09 (0.96, 4.54) | 1.00 | 1.61 (0.69, 3.77) | 1.13 (0.50, 2.54) | 0.99 (0.37, 2.63) | 0.62 (0.17, 2.24) | 0.145 |  |
| Women |  |  |  |  |  |  |  |  |  |  |
| No. of cases | 158 | 19 | 21 | 38 | 19 | 36 | 14 | 11 |  |  |
| Cases/PYs (1/1000) | 0.07 | 0.07 | 0.12 | 0.07 | 0.06 | 0.07 | 0.04 | 0.04 |  |  |
| HR (95% CI) |  | 0.70 (0.39, 1.25) | 1.41 (0.82, 2.42) | 1.00 | 0.95 (0.55, 1.65) | 1.02 (0.64, 1.62) | 0.63 (0.34, 1.18) | 0.63 (0.31, 1.27) | 0.262 |  |
| Hip fracture |  |  |  |  |  |  |  |  |  | 0.144 |
| Men |  |  |  |  |  |  |  |  |  |  |
| No. of cases | 401 | 123 | 39 | 84 | 31 | 60 | 22 | 42 |  |  |
| Cases/PYs (1/1000) | 0.23 | 0.48 | 0.28 | 0.24 | 0.18 | 0.19 | 0.09 | 0.17 |  |  |
| HR (95% CI) |  | 1.38 (1.03, 1.86) | 1.01 (0.69, 1.47) | 1.00 | 0.77 (0.51, 1.17) | 0.77 (0.55, 1.07) | 0.36 (0.22, 0.57) | 0.61 (0.41, 0.91) | <0.001 |  |
| Women |  |  |  |  |  |  |  |  |  |  |
| No. of cases | 821 | 214 | 76 | 164 | 74 | 120 | 93 | 80 |  |  |
| Cases/PYs (1/1000) | 0.34 | 0.81 | 0.43 | 0.32 | 0.25 | 0.23 | 0.27 | 0.28 |  |  |
| HR (95% CI) |  | 1.42 (1.14, 1.76) | 1.08 (0.82, 1.42) | 1.00 | 0.86 (0.65, 1.13) | 0.69 (0.54, 0.87) | 0.70 (0.54, 0.92) | 0.59 (0.44, 0.78) | <0.001 |  |
| Other central body fracture |  |  |  |  |  |  |  |  |  | 0.464 |
| Men |  |  |  |  |  |  |  |  |  |  |
| No. of cases | 782 | 176 | 89 | 162 | 70 | 131 | 88 | 66 |  |  |
| Cases/PYs (1/1000) | 0.45 | 0.69 | 0.63 | 0.47 | 0.42 | 0.41 | 0.36 | 0.27 |  |  |
| HR (95% CI) |  | 1.01 (0.80, 1.27) | 1.08 (0.84, 1.41) | 1.00 | 1.03 (0.78, 1.37) | 1.11 (0.88, 1.41) | 1.14 (0.88, 1.49) | 1.05 (0.77, 1.41) | 0.521 |  |
| Women |  |  |  |  |  |  |  |  |  |  |
| No. of cases | 663 | 128 | 61 | 118 | 82 | 132 | 73 | 69 |  |  |
| Cases/PYs (1/1000) | 0.28 | 0.49 | 0.35 | 0.23 | 0.28 | 0.25 | 0.22 | 0.25 |  |  |
| HR (95% CI) |  | 1.39 (1.07, 1.81) | 1.24 (0.91, 1.69) | 1.00 | 1.39 (1.05, 1.85) | 1.31 (1.02, 1.69) | 1.15 (0.85, 1.55) | 1.44 (1.06, 1.97) | 0.697 |  |
| Other fracture |  |  |  |  |  |  |  |  |  | 0.713 |
| Men |  |  |  |  |  |  |  |  |  |  |
| No. of cases | 1,050 | 206 | 92 | 249 | 106 | 185 | 119 | 93 |  |  |
| Cases/PYs (1/1000) | 0.61 | 0.81 | 0.66 | 0.72 | 0.63 | 0.58 | 0.49 | 0.38 |  |  |
| HR (95% CI) |  | 1.23 (1.01, 1.50) | 0.97 (0.76, 1.24) | 1.00 | 0.96 (0.76, 1.20) | 0.97 (0.80, 1.17) | 0.92 (0.73, 1.15) | 0.80 (0.63, 1.03) | 0.005 |  |
| Women |  |  |  |  |  |  |  |  |  |  |
| No. of cases | 1,056 | 139 | 68 | 225 | 119 | 251 | 133 | 121 |  |  |
| Cases/PYs (1/1000) | 0.44 | 0.53 | 0.39 | 0.44 | 0.40 | 0.47 | 0.39 | 0.43 |  |  |
| HR (95% CI) |  | 1.05 (0.84, 1.32) | 0.86 (0.65, 1.13) | 1.00 | 0.98 (0.78, 1.22) | 1.12 (0.94, 1.35) | 0.93 (0.75, 1.16) | 0.94 (0.74, 1.18) | 0.694 |  |

HR, hazard ratio; CI, confidence interval; PYs, person-years.

Multivariable models were adjusted for the same set of covariates as in model 3 of Table 2, except for sex. Models in women additionally included menopausal status (premenopausal, perimenopausal, postmenopausal with age at menopause <45, 45-49, ≥50 years, or missing values).

Appendix table 4. Association between weight change from age 25 to baseline and risk of any fracture according to baseline factors.

|  | Weight change from age 25 to baseline, kg | | | | | | | | P_interaction_ |
| --- | --- | --- | --- | --- | --- | --- | --- | --- | --- |
| ≤-2.5 | |  | -2.4-2.4 | |  | ≥2.5 | |  |  |
| Cases | HR (95%CI) |  | Cases | HR |  | Cases | HR (95%CI) |  |  |
| Age at baseline (yr) |  |  |  |  |  |  |  |  | 0.249 |
| <65 | 2,514 | 1.03 (0.97, 1.09) |  | 2,287 | 1.00 |  | 5,608 | 0.98 (0.93, 1.03) |  |
| ≥65 | 1,025 | 1.11 (0.99, 1.24) |  | 481 | 1.00 |  | 1,150 | 0.96 (0.86, 1.07) |  |
| Residence |  |  |  |  |  |  |  |  | 0.003 |
| Rural | 2,721 | 1.08 (1.02, 1.15) |  | 2,057 | 1.00 |  | 4,335 | 1.00 (0.94, 1.05) |  |
| Urban | 818 | 1.07 (0.96, 1.18) |  | 711 | 1.00 |  | 2,423 | 0.87 (0.80, 0.95) |  |
| BMI at age 25^*^ (kg/m^2^) |  |  |  |  |  |  |  |  | 0.176 |
| <24.0 | 1,953 | 1.01 (0.95, 1.08) |  | 2,218 | 1.00 |  | 5,995 | 0.97 (0.92, 1.02) |  |
| ≥24.0 | 1,586 | 1.14 (1.03, 1.25) |  | 550 | 1.00 |  | 763 | 1.03 (0.92, 1.15) |  |
| BMI at baseline (kg/m^2^) |  |  |  |  |  |  |  |  | 0.186 |
| <24.0 | 3,178 | 1.03 (0.97, 1.09) |  | 2,213 | 1.00 |  | 2,354 | 0.98 (0.92, 1.04) |  |
| ≥24.0 | 361 | 1.18 (1.03, 1.35) |  | 555 | 1.00 |  | 4,404 | 1.04 (0.95, 1.13) |  |
| Smoking |  |  |  |  |  |  |  |  | 0.145 |
| Never | 2,073 | 1.04 (0.98, 1.11) |  | 1,827 | 1.00 |  | 4,821 | 0.99 (0.94, 1.05) |  |
| Former or current | 1,466 | 1.07 (0.98, 1.17) |  | 941 | 1.00 |  | 1,937 | 0.93 (0.86, 1.01) |  |
| Physical activity^†^ |  |  |  |  |  |  |  |  | 0.904 |
| Low | 1,030 | 1.06 (0.96, 1.17) |  | 753 | 1.00 |  | 2,309 | 0.97 (0.89, 1.05) |  |
| Middle | 1,111 | 1.01 (0.92, 1.11) |  | 885 | 1.00 |  | 2,118 | 0.97 (0.89, 1.05) |  |
| High | 1,398 | 1.07 (0.98, 1.16) |  | 1,130 | 1.00 |  | 2,331 | 0.99 (0.92, 1.07) |  |
| Intake of supplementary calcium, iron or zinc |  |  |  |  |  |  |  |  | 0.144 |
| Yes | 252 | 1.34 (1.09, 1.64) |  | 166 | 1.00 |  | 591 | 1.07 (0.90, 1.28) |  |
| No | 3,287 | 1.03 (0.98, 1.09) |  | 2,602 | 1.00 |  | 6,167 | 0.97 (0.93, 1.02) |  |
| Intake of dairy products |  |  |  |  |  |  |  |  | 0.254 |
| At least once per week | 419 | 1.13 (0.97, 1.31) |  | 334 | 1.00 |  | 1,223 | 0.96 (0.85, 1.09) |  |
| Less than weekly | 3,120 | 1.04 (0.98, 1.10) |  | 2,434 | 1.00 |  | 5,535 | 0.99 (0.94, 1.03) |  |
| Menopausal status (in women^)^ |  |  |  |  |  |  |  |  | 0.082 |
| Premenopausal or perimenopausal | 346 | 0.92 (0.80, 1.05) |  | 568 | 1.00 |  | 1,411 | 1.00 (0.91, 1.11) |  |
| Postmenopausal | 1,634 | 1.07 (0.99, 1.16) |  | 1,087 | 1.00 |  | 3,009 | 1.01 (0.94, 1.09) |  |

HR indicates hazard ratios; CI confidence interval; MET, metabolic equivalent of task.

Multivariable models were adjusted for the same set of covariates as in model 3 of Table 2.

^*^BMI at age 25 was calculated using self-reported weight at age 25 and measured height at baseline

^†^Physical activity (MET h/day) was categorized using tertile cut-offs by sex (for men: <13.17, 13.17 to <28.25, ≥28.25; for women: <12.33, 12.33 to <23.30, ≥23.30).

Appendix table 5. Association between weight change from age 25 to baseline and risk of upper limb fracture according to baseline factors.

|  | Weight change from age 25 to baseline, kg | | | | | | | | P_interaction_ |
| --- | --- | --- | --- | --- | --- | --- | --- | --- | --- |
| ≤-2.5 | |  | -2.4-2.4 | |  | ≥2.5 | |  |  |
| Cases | HR (95%CI) |  | Cases | HR |  | Cases | HR (95%CI) |  |  |
| Age at baseline (yr) |  |  |  |  |  |  |  |  | 0.301 |
| <65 | 657 | 0.90 (0.81, 1.01) |  | 655 | 1.00 |  | 1,565 | 0.99 (0.90, 1.09) |  |
| ≥65 | 204 | 0.95 (0.74, 1.21) |  | 108 | 1.00 |  | 222 | 0.85 (0.67, 1.08) |  |
| Residence |  |  |  |  |  |  |  |  | 0.412 |
| Rural | 684 | 0.95 (0.85, 1.07) |  | 582 | 1.00 |  | 1,187 | 0.99 (0.89, 1.09) |  |
| Urban | 177 | 1.01 (0.81, 1.25) |  | 181 | 1.00 |  | 600 | 0.81 (0.68, 0.96) |  |
| BMI at age 25^*^ (kg/m^2^) |  |  |  |  |  |  |  |  | 0.676 |
| <24.0 | 461 | 0.89 (0.79, 1.01) |  | 593 | 1.00 |  | 1,589 | 0.99 (0.90, 1.09) |  |
| ≥24.0 | 400 | 0.94 (0.78, 1.12) |  | 170 | 1.00 |  | 198 | 0.92 (0.75, 1.13) |  |
| BMI at baseline (kg/m^2^) |  |  |  |  |  |  |  |  | 0.220 |
| <24.0 | 765 | 0.92 (0.82, 1.02) |  | 597 | 1.00 |  | 647 | 0.99 (0.88, 1.11) |  |
| ≥24.0 | 96 | 1.06 (0.82, 1.36) |  | 166 | 1.00 |  | 1,140 | 0.93 (0.79, 1.10) |  |
| Smoking |  |  |  |  |  |  |  |  | 0.716 |
| Never | 556 | 0.90 (0.79, 1.01) |  | 550 | 1.00 |  | 1,341 | 0.97 (0.87, 1.07) |  |
| Former or current | 305 | 0.94 (0.79, 1.14) |  | 213 | 1.00 |  | 446 | 0.95 (0.80, 1.13) |  |
| Physical activity^†^ |  |  |  |  |  |  |  |  | 0.489 |
| Low | 177 | 0.89 (0.71, 1.12) |  | 157 | 1.00 |  | 509 | 1.00 (0.83, 1.21) |  |
| Middle | 276 | 0.84 (0.70, 1.00) |  | 258 | 1.00 |  | 578 | 0.94 (0.81, 1.10) |  |
| High | 408 | 0.95 (0.82, 1.10) |  | 348 | 1.00 |  | 700 | 0.99 (0.86, 1.13) |  |
| Intake of supplementary calcium, iron or zinc |  |  |  |  |  |  |  |  | 0.129 |
| Yes | 65 | 1.23 (0.83, 1.84) |  | 44 | 1.00 |  | 141 | 1.02 (0.72, 1.46) |  |
| No | 796 | 0.89 (0.80, 0.99) |  | 719 | 1.00 |  | 1,646 | 0.98 (0.89, 1.07) |  |
| Intake of dairy products |  |  |  |  |  |  |  |  | 0.387 |
| At least once per week | 97 | 0.87 (0.65, 1.16) |  | 104 | 1.00 |  | 326 | 0.87 (0.69, 1.09) |  |
| Less than weekly | 764 | 0.91 (0.82, 1.02) |  | 659 | 1.00 |  | 1,461 | 1.00 (0.91, 1.10) |  |
| Menopausal status (in women^)^ |  |  |  |  |  |  |  |  | 0.844 |
| Premenopausal or perimenopausal | 102 | 0.86 (0.66, 1.10) |  | 167 | 1.00 |  | 398 | 1.03 (0.85, 1.24) |  |
| Postmenopausal | 481 | 0.92 (0.80, 1.06) |  | 361 | 1.00 |  | 895 | 0.96 (0.85, 1.09) |  |

HR indicates hazard ratios; CI confidence interval; MET, metabolic equivalent of task.

Multivariable models were adjusted for the same set of covariates as in model 3 of Table 2.

^*^BMI at age 25 was calculated using self-reported weight at age 25 and measured height at baseline

^†^Physical activity (MET h/day) was categorized using tertile cut-offs by sex (for men: <13.17, 13.17 to <28.25, ≥28.25; for women: <12.33, 12.33 to <23.30, ≥23.30).

Appendix table 6. Association between weight change from age 25 to baseline and risk of lower limb fracture according to baseline factors.

|  | Weight change from age 25 to baseline, kg | | | | | | | | P_interaction_ |
| --- | --- | --- | --- | --- | --- | --- | --- | --- | --- |
| ≤-2.5 | |  | -2.4-2.4 | |  | ≥2.5 | |  |  |
| Cases | HR (95%CI) |  | Cases | HR |  | Cases | HR (95%CI) |  |  |
| Age at baseline (yr) |  |  |  |  |  |  |  |  | 0.068 |
| <65 | 751 | 1.03 (0.93, 1.15) |  | 688 | 1.00 |  | 1,587 | 0.90 (0.82, 0.99) |  |
| ≥65 | 268 | 1.38 (1.09, 1.76) |  | 96 | 1.00 |  | 250 | 1.13 (0.88, 1.44) |  |
| Residence |  |  |  |  |  |  |  |  | 0.350 |
| Rural | 775 | 1.21 (1.08, 1.36) |  | 554 | 1.00 |  | 1,128 | 0.93 (0.84, 1.03) |  |
| Urban | 244 | 1.01 (0.84, 1.22) |  | 230 | 1.00 |  | 709 | 0.83 (0.72, 0.97) |  |
| BMI at age 25^*^ (kg/m^2^) |  |  |  |  |  |  |  |  | 0.560 |
| <24.0 | 567 | 1.05 (0.94, 1.18) |  | 620 | 1.00 |  | 1,644 | 0.96 (0.87, 1.05) |  |
| ≥24.0 | 452 | 1.09 (0.91, 1.31) |  | 164 | 1.00 |  | 193 | 0.87 (0.70, 1.07) |  |
| BMI at baseline (kg/m^2^) |  |  |  |  |  |  |  |  | 0.982 |
| <24.0 | 921 | 1.08 (0.98, 1.20) |  | 617 | 1.00 |  | 626 | 0.94 (0.84, 1.05) |  |
| ≥24.0 | 98 | 1.07 (0.83, 1.37) |  | 167 | 1.00 |  | 1,211 | 0.93 (0.79, 1.10) |  |
| Smoking |  |  |  |  |  |  |  |  | 0.185 |
| Never | 596 | 1.12 (0.99, 1.27) |  | 499 | 1.00 |  | 1,263 | 0.96 (0.86, 1.07) |  |
| Former or current | 423 | 1.04 (0.89, 1.22) |  | 285 | 1.00 |  | 574 | 0.87 (0.75, 1.00) |  |
| Physical activity^†^ |  |  |  |  |  |  |  |  | 0.298 |
| Low | 266 | 1.25 (1.02, 1.53) |  | 162 | 1.00 |  | 526 | 1.02 (0.85, 1.22) |  |
| Middle | 319 | 0.98 (0.83, 1.16) |  | 274 | 1.00 |  | 588 | 0.86 (0.74, 0.99) |  |
| High | 434 | 1.10 (0.95, 1.27) |  | 348 | 1.00 |  | 723 | 0.97 (0.85, 1.10) |  |
| Intake of supplementary calcium, iron or zinc |  |  |  |  |  |  |  |  | 0.301 |
| Yes | 68 | 1.46 (0.97, 2.20) |  | 39 | 1.00 |  | 137 | 1.10 (0.75, 1.59) |  |
| No | 951 | 1.07 (0.97, 1.18) |  | 745 | 1.00 |  | 1,700 | 0.93 (0.85, 1.01) |  |
| Intake of dairy products |  |  |  |  |  |  |  |  | 0.671 |
| At least once per week | 99 | 1.18 (0.86, 1.61) |  | 77 | 1.00 |  | 307 | 1.03 (0.79, 1.33) |  |
| Less than weekly | 920 | 1.08 (0.97, 1.19) |  | 707 | 1.00 |  | 1,530 | 0.93 (0.85, 1.02) |  |
| Menopausal status (in women^)^ |  |  |  |  |  |  |  |  | 0.028 |
| Premenopausal or perimenopausal | 107 | 0.84 (0.66, 1.07) |  | 192 | 1.00 |  | 445 | 0.93 (0.78, 1.11) |  |
| Postmenopausal | 438 | 1.22 (1.04, 1.43) |  | 255 | 1.00 |  | 706 | 1.02 (0.88, 1.19) |  |

HR indicates hazard ratios; CI confidence interval; MET, metabolic equivalent of task.

Multivariable models were adjusted for the same set of covariates as in model 3 of Table 2.

^*^BMI at age 25 was calculated using self-reported weight at age 25 and measured height at baseline

^†^Physical activity (MET h/day) was categorized using tertile cut-offs by sex (for men: <13.17, 13.17 to <28.25, ≥28.25; for women: <12.33, 12.33 to <23.30, ≥23.30).

Appendix table 7. Association between weight change from age 25 to baseline and risk of spine fracture according to baseline factors.

|  | Weight change from age 25 to baseline, kg | | | | | | | | P_interaction_ |
| --- | --- | --- | --- | --- | --- | --- | --- | --- | --- |
| ≤-2.5 | |  | -2.4-2.4 | |  | ≥2.5 | |  |  |
| Cases | HR (95%CI) |  | Cases | HR |  | Cases | HR (95%CI) |  |  |
| Age at baseline (yr) |  |  |  |  |  |  |  |  | 0.573 |
| <65 | 311 | 0.97 (0.82, 1.15) |  | 278 | 1.00 |  | 845 | 1.14 (0.99, 1.32) |  |
| ≥65 | 172 | 1.07 (0.82, 1.39) |  | 91 | 1.00 |  | 276 | 1.06 (0.82, 1.35) |  |
| Residence |  |  |  |  |  |  |  |  | 0.826 |
| Rural | 324 | 1.04 (0.88, 1.24) |  | 239 | 1.00 |  | 558 | 1.11 (0.95, 1.30) |  |
| Urban | 159 | 1.10 (0.87, 1.39) |  | 130 | 1.00 |  | 563 | 1.06 (0.87, 1.29) |  |
| BMI at age 25^*^ (kg/m^2^) |  |  |  |  |  |  |  |  | 0.177 |
| <24.0 | 261 | 0.95 (0.81, 1.13) |  | 305 | 1.00 |  | 1,007 | 1.08 (0.94, 1.23) |  |
| ≥24.0 | 222 | 1.27 (0.96, 1.68) |  | 64 | 1.00 |  | 114 | 1.43 (1.05, 1.95) |  |
| BMI at baseline (kg/m^2^) |  |  |  |  |  |  |  |  | 0.031 |
| <24.0 | 423 | 0.92 (0.79, 1.07) |  | 302 | 1.00 |  | 374 | 1.06 (0.90, 1.23) |  |
| ≥24.0 | 60 | 1.44 (1.02, 2.05) |  | 67 | 1.00 |  | 747 | 1.54 (1.19, 1.98) |  |
| Smoking |  |  |  |  |  |  |  |  | 0.058 |
| Never | 303 | 0.94 (0.79, 1.11) |  | 276 | 1.00 |  | 903 | 1.15 (1.00, 1.32) |  |
| Former or current | 180 | 1.20 (0.92, 1.56) |  | 93 | 1.00 |  | 218 | 1.03 (0.80, 1.33) |  |
| Physical activity^†^ |  |  |  |  |  |  |  |  | 0.759 |
| Low | 167 | 1.10 (0.86, 1.40) |  | 118 | 1.00 |  | 445 | 1.12 (0.91, 1.38) |  |
| Middle | 148 | 1.00 (0.77, 1.29) |  | 111 | 1.00 |  | 358 | 1.23 (0.99, 1.54) |  |
| High | 168 | 0.96 (0.76, 1.22) |  | 140 | 1.00 |  | 318 | 1.06 (0.86, 1.30) |  |
| Intake of supplementary calcium, iron or zinc |  |  |  |  |  |  |  |  | 0.310 |
| Yes | 35 | 1.12 (0.67, 1.87) |  | 28 | 1.00 |  | 140 | 1.33 (0.87, 2.04) |  |
| No | 448 | 1.00 (0.87, 1.16) |  | 341 | 1.00 |  | 981 | 1.10 (0.97, 1.25) |  |
| Intake of dairy products |  |  |  |  |  |  |  |  | 0.079 |
| At least once per week | 89 | 1.31 (0.93, 1.84) |  | 59 | 1.00 |  | 269 | 1.11 (0.83, 1.49) |  |
| Less than weekly | 394 | 0.97 (0.83, 1.13) |  | 310 | 1.00 |  | 852 | 1.13 (0.99, 1.30) |  |
| Menopausal status (in women^)^ |  |  |  |  |  |  |  |  | 0.918 |
| Premenopausal or perimenopausal | 39 | 1.03 (0.68, 1.56) |  | 61 | 1.00 |  | 172 | 1.00 (0.74, 1.35) |  |
| Postmenopausal | 274 | 0.92 (0.77, 1.11) |  | 206 | 1.00 |  | 688 | 1.18 (1.00, 1.38) |  |

HR indicates hazard ratios; CI confidence interval; MET, metabolic equivalent of task.

Multivariable models were adjusted for the same set of covariates as in model 3 of Table 2.

^*^BMI at age 25 was calculated using self-reported weight at age 25 and measured height at baseline

^†^Physical activity (MET h/day) was categorized using tertile cut-offs by sex (for men: <13.17, 13.17 to <28.25, ≥28.25; for women: <12.33, 12.33 to <23.30, ≥23.30).

Appendix table 8. Association between weight change from age 25 to baseline and risk of pelvis fracture according to baseline factors.

|  | Weight change from age 25 to baseline, kg | | | | | | | | P_interaction_ |
| --- | --- | --- | --- | --- | --- | --- | --- | --- | --- |
| ≤-2.5 | |  | -2.4-2.4 | |  | ≥2.5 | |  |  |
| Cases | HR (95%CI) |  | Cases | HR |  | Cases | HR (95%CI) |  |  |
| Age at baseline (yr) |  |  |  |  |  |  |  |  | 0.289 |
| <65 | 57 | 1.28 (0.84, 1.93) |  | 43 | 1.00 |  | 92 | 0.93 (0.64, 1.34) |  |
| ≥65 | 12 | 0.79 (0.31, 2.02) |  | 8 | 1.00 |  | 17 | 0.94 (0.39, 2.27) |  |
| Residence |  |  |  |  |  |  |  |  | 0.563 |
| Rural | 60 | 1.19 (0.79, 1.78) |  | 45 | 1.00 |  | 91 | 0.90 (0.62, 1.30) |  |
| Urban | 9 | 1.44 (0.49, 4.19) |  | 6 | 1.00 |  | 18 | 0.73 (0.28, 1.92) |  |
| BMI at age 25^*^ (kg/m^2^) |  |  |  |  |  |  |  |  | 0.118 |
| <24.0 | 41 | 0.96 (0.63, 1.48) |  | 46 | 1.00 |  | 95 | 0.84 (0.59, 1.21) |  |
| ≥24.0 | 28 | 2.35 (0.89, 6.16) |  | 5 | 1.00 |  | 14 | 2.10 (0.75, 5.87) |  |
| BMI at baseline (kg/m^2^) |  |  |  |  |  |  |  |  | 0.754 |
| <24.0 | 63 | 0.97 (0.65, 1.44) |  | 44 | 1.00 |  | 46 | 1.00 (0.66, 1.52) |  |
| ≥24.0 | 6 | 1.65 (0.55, 4.95) |  | 7 | 1.00 |  | 63 | 1.28 (0.58, 2.82) |  |
| Smoking |  |  |  |  |  |  |  |  | 0.469 |
| Never | 39 | 0.92 (0.58, 1.47) |  | 39 | 1.00 |  | 82 | 0.87 (0.58, 1.29) |  |
| Former or current | 30 | 1.86 (0.92, 3.74) |  | 12 | 1.00 |  | 27 | 1.06 (0.53, 2.12) |  |
| Physical activity^†^ |  |  |  |  |  |  |  |  | 0.212 |
| Low | 10 | 0.60 (0.24, 1.46) |  | 11 | 1.00 |  | 31 | 1.08 (0.53, 2.20) |  |
| Middle | 29 | 1.94 (0.99, 3.79) |  | 14 | 1.00 |  | 29 | 0.81 (0.42, 1.56) |  |
| High | 30 | 1.02 (0.59, 1.75) |  | 26 | 1.00 |  | 49 | 0.89 (0.55, 1.45) |  |
| Intake of supplementary calcium, iron or zinc |  |  |  |  |  |  |  |  | 0.511 |
| Yes | 7 | 1.70 (0.40, 7.20) |  | 3 | 1.00 |  | 8 | 0.86 (0.21, 3.55) |  |
| No | 62 | 1.10 (0.74, 1.63) |  | 48 | 1.00 |  | 101 | 0.92 (0.65, 1.31) |  |
| Intake of dairy products |  |  |  |  |  |  |  |  | 0.977 |
| At least once per week | 6 | 1.02 (0.29, 3.62) |  | 5 | 1.00 |  | 14 | 0.69 (0.24, 2.03) |  |
| Less than weekly | 63 | 1.16 (0.78, 1.73) |  | 46 | 1.00 |  | 95 | 0.94 (0.65, 1.35) |  |
| Menopausal status (in women^)^ |  |  |  |  |  |  |  |  | 0.382 |
| Premenopausal or perimenopausal | 13 | 1.42 (0.65, 3.09) |  | 14 | 1.00 |  | 33 | 1.10 (0.58, 2.09) |  |
| Postmenopausal | 27 | 0.79 (0.45, 1.41) |  | 24 | 1.00 |  | 47 | 0.71 (0.42, 1.18) |  |

HR indicates hazard ratios; CI confidence interval; MET, metabolic equivalent of task.

Multivariable models were adjusted for the same set of covariates as in model 3 of Table 2.

^*^BMI at age 25 was calculated using self-reported weight at age 25 and measured height at baseline

^†^Physical activity (MET h/day) was categorized using tertile cut-offs by sex (for men: <13.17, 13.17 to <28.25, ≥28.25; for women: <12.33, 12.33 to <23.30, ≥23.30).

Appendix table 9. Association between weight change from age 25 to baseline and risk of hip fracture according to baseline factors.

|  | Weight change from age 25 to baseline, kg | | | | | | | | P_interaction_ |
| --- | --- | --- | --- | --- | --- | --- | --- | --- | --- |
| ≤-2.5 | |  | -2.4-2.4 | |  | ≥2.5 | |  |  |
| Cases | HR (95%CI) |  | Cases | HR |  | Cases | HR (95%CI) |  |  |
| Age at baseline (yr) |  |  |  |  |  |  |  |  | 0.819 |
| <65 | 188 | 1.26 (0.99, 1.59) |  | 129 | 1.00 |  | 283 | 0.69 (0.56, 0.86) |  |
| ≥65 | 264 | 1.22 (0.97, 1.53) |  | 119 | 1.00 |  | 239 | 0.67 (0.53, 0.85) |  |
| Residence |  |  |  |  |  |  |  |  | 0.263 |
| Rural | 255 | 1.34 (1.07, 1.68) |  | 126 | 1.00 |  | 205 | 0.74 (0.59, 0.93) |  |
| Urban | 197 | 1.30 (1.03, 1.64) |  | 122 | 1.00 |  | 317 | 0.60 (0.48, 0.75) |  |
| BMI at age 25^*^ (kg/m^2^) |  |  |  |  |  |  |  |  | 0.579 |
| <24.0 | 240 | 1.21 (0.99, 1.46) |  | 196 | 1.00 |  | 461 | 0.66 (0.56, 0.79) |  |
| ≥24.0 | 212 | 1.38 (1.01, 1.88) |  | 52 | 1.00 |  | 61 | 0.85 (0.59, 1.24) |  |
| BMI at baseline (kg/m^2^) |  |  |  |  |  |  |  |  | 0.130 |
| <24.0 | 400 | 1.12 (0.94, 1.33) |  | 203 | 1.00 |  | 197 | 0.79 (0.65, 0.96) |  |
| ≥24.0 | 52 | 1.73 (1.16, 2.58) |  | 45 | 1.00 |  | 325 | 0.87 (0.63, 1.19) |  |
| Smoking |  |  |  |  |  |  |  |  | 0.508 |
| Never | 286 | 1.26 (1.03, 1.53) |  | 176 | 1.00 |  | 402 | 0.72 (0.60, 0.87) |  |
| Former or current | 166 | 1.27 (0.95, 1.70) |  | 72 | 1.00 |  | 120 | 0.58 (0.43, 0.79) |  |
| Physical activity^†^ |  |  |  |  |  |  |  |  | 0.607 |
| Low | 231 | 1.29 (1.03, 1.63) |  | 116 | 1.00 |  | 285 | 0.70 (0.56, 0.87) |  |
| Middle | 131 | 1.19 (0.88, 1.60) |  | 79 | 1.00 |  | 145 | 0.63 (0.48, 0.84) |  |
| High | 90 | 1.29 (0.90, 1.84) |  | 53 | 1.00 |  | 92 | 0.74 (0.52, 1.05) |  |
| Intake of supplementary calcium, iron or zinc |  |  |  |  |  |  |  |  | 0.700 |
| Yes | 38 | 1.17 (0.70, 1.95) |  | 27 | 1.00 |  | 73 | 0.68 (0.42, 1.09) |  |
| No | 414 | 1.27 (1.07, 1.51) |  | 221 | 1.00 |  | 449 | 0.68 (0.58, 0.81) |  |
| Intake of dairy products |  |  |  |  |  |  |  |  | 0.614 |
| At least once per week | 92 | 1.52 (1.06, 2.17) |  | 51 | 1.00 |  | 165 | 0.73 (0.53, 1.02) |  |
| Less than weekly | 360 | 1.21 (1.01, 1.45) |  | 197 | 1.00 |  | 357 | 0.68 (0.56, 0.81) |  |
| Menopausal status (in women^)^ |  |  |  |  |  |  |  |  | 0.701 |
| Premenopausal or perimenopausal | 19 | 0.99 (0.54, 1.81) |  | 27 | 1.00 |  | 50 | 0.69 (0.42, 1.12) |  |
| Postmenopausal | 271 | 1.33 (1.07, 1.65) |  | 137 | 1.00 |  | 317 | 0.71 (0.58, 0.88) |  |

HR indicates hazard ratios; CI confidence interval; MET, metabolic equivalent of task.

Multivariable models were adjusted for the same set of covariates as in model 3 of Table 2.

^*^BMI at age 25 was calculated using self-reported weight at age 25 and measured height at baseline

^†^Physical activity (MET h/day) was categorized using tertile cut-offs by sex (for men: <13.17, 13.17 to <28.25, ≥28.25; for women: <12.33, 12.33 to <23.30, ≥23.30).

Appendix table 10. Association between weight change from age 25 to baseline and risk of other central body fracture according to baseline factors.

|  | Weight change from age 25 to baseline, kg | | | | | | | | P_interaction_ |
| --- | --- | --- | --- | --- | --- | --- | --- | --- | --- |
| ≤-2.5 | |  | -2.4-2.4 | |  | ≥2.5 | |  |  |
| Cases | HR (95%CI) |  | Cases | HR |  | Cases | HR (95%CI) |  |  |
| Age at baseline (yr) |  |  |  |  |  |  |  |  | <0.001 |
| <65 | 355 | 1.25 (1.05, 1.48) |  | 234 | 1.00 |  | 594 | 1.14 (0.98, 1.34) |  |
| ≥65 | 99 | 0.88 (0.61, 1.27) |  | 46 | 1.00 |  | 117 | 1.42 (0.99, 2.02) |  |
| Residence |  |  |  |  |  |  |  |  | 0.053 |
| Rural | 410 | 1.26 (1.06, 1.49) |  | 239 | 1.00 |  | 524 | 1.11 (0.95, 1.29) |  |
| Urban | 44 | 1.01 (0.65, 1.56) |  | 41 | 1.00 |  | 187 | 1.27 (0.90, 1.80) |  |
| BMI at age 25^*^ (kg/m^2^) |  |  |  |  |  |  |  |  | 0.039 |
| <24.0 | 271 | 1.12 (0.94, 1.34) |  | 242 | 1.00 |  | 629 | 1.08 (0.93, 1.26) |  |
| ≥24.0 | 183 | 1.61 (1.13, 2.29) |  | 38 | 1.00 |  | 82 | 1.83 (1.24, 2.69) |  |
| BMI at baseline (kg/m^2^) |  |  |  |  |  |  |  |  | 0.020 |
| <24.0 | 423 | 1.14 (0.97, 1.34) |  | 242 | 1.00 |  | 237 | 1.03 (0.86, 1.23) |  |
| ≥24.0 | 31 | 1.48 (0.92, 2.39) |  | 38 | 1.00 |  | 474 | 1.66 (1.19, 2.32) |  |
| Smoking |  |  |  |  |  |  |  |  | 0.220 |
| Never | 214 | 1.30 (1.04, 1.62) |  | 142 | 1.00 |  | 419 | 1.30 (1.07, 1.58) |  |
| Former or current | 240 | 1.03 (0.83, 1.28) |  | 138 | 1.00 |  | 292 | 1.07 (0.87, 1.32) |  |
| Physical activity^†^ |  |  |  |  |  |  |  |  | 0.063 |
| Low | 83 | 1.00 (0.69, 1.43) |  | 52 | 1.00 |  | 173 | 1.25 (0.91, 1.73) |  |
| Middle | 139 | 1.19 (0.89, 1.59) |  | 76 | 1.00 |  | 235 | 1.50 (1.15, 1.95) |  |
| High | 232 | 1.22 (0.99, 1.51) |  | 152 | 1.00 |  | 303 | 1.02 (0.84, 1.25) |  |
| Intake of supplementary calcium, iron or zinc |  |  |  |  |  |  |  |  | 0.070 |
| Yes | 24 | 2.53 (1.11, 5.75) |  | 8 | 1.00 |  | 54 | 2.30 (1.07, 4.95) |  |
| No | 430 | 1.12 (0.95, 1.31) |  | 272 | 1.00 |  | 657 | 1.15 (0.99, 1.33) |  |
| Intake of dairy products |  |  |  |  |  |  |  |  | 0.436 |
| At least once per week | 25 | 1.06 (0.55, 2.01) |  | 17 | 1.00 |  | 92 | 1.60 (0.94, 2.74) |  |
| Less than weekly | 429 | 1.16 (0.99, 1.37) |  | 263 | 1.00 |  | 619 | 1.16 (1.00, 1.34) |  |
| Menopausal status (in women^)^ |  |  |  |  |  |  |  |  | 0.948 |
| Premenopausal or perimenopausal | 41 | 1.26 (0.81, 1.94) |  | 45 | 1.00 |  | 128 | 1.26 (0.89, 1.79) |  |
| Postmenopausal | 148 | 1.37 (1.03, 1.83) |  | 73 | 1.00 |  | 228 | 1.35 (1.03, 1.77) |  |

HR indicates hazard ratios; CI confidence interval; MET, metabolic equivalent of task.

Multivariable models were adjusted for the same set of covariates as in model 3 of Table 2.

^*^BMI at age 25 was calculated using self-reported weight at age 25 and measured height at baseline

^†^Physical activity (MET h/day) was categorized using tertile cut-offs by sex (for men: <13.17, 13.17 to <28.25, ≥28.25; for women: <12.33, 12.33 to <23.30, ≥23.30).

Appendix table 11. Association between weight change from age 25 to baseline and risk of other fracture according to baseline factors.

|  | Weight change from age 25 to baseline, kg | | | | | | | | P_interaction_ |
| --- | --- | --- | --- | --- | --- | --- | --- | --- | --- |
| ≤-2.5 | |  | -2.4-2.4 | |  | ≥2.5 | |  |  |
| Cases | HR (95%CI) |  | Cases | HR |  | Cases | HR (95%CI) |  |  |
| Age at baseline (yr) |  |  |  |  |  |  |  |  | 0.466 |
| <65 | 403 | 1.02 (0.89, 1.18) |  | 420 | 1.00 |  | 992 | 0.97 (0.86, 1.09) |  |
| ≥65 | 102 | 1.01 (0.72, 1.43) |  | 54 | 1.00 |  | 135 | 1.10 (0.79, 1.53) |  |
| Residence |  |  |  |  |  |  |  |  | 0.061 |
| Rural | 452 | 0.95 (0.82, 1.09) |  | 429 | 1.00 |  | 970 | 1.03 (0.92, 1.16) |  |
| Urban | 53 | 0.96 (0.64, 1.45) |  | 45 | 1.00 |  | 157 | 0.98 (0.70, 1.39) |  |
| BMI at age 25^*^ (kg/m^2^) |  |  |  |  |  |  |  |  | 0.881 |
| <24.0 | 281 | 1.02 (0.87, 1.19) |  | 379 | 1.00 |  | 978 | 0.97 (0.86, 1.10) |  |
| ≥24.0 | 224 | 1.08 (0.84, 1.38) |  | 95 | 1.00 |  | 149 | 1.00 (0.77, 1.30) |  |
| BMI at baseline (kg/m^2^) |  |  |  |  |  |  |  |  | 0.482 |
| <24.0 | 462 | 1.08 (0.94, 1.24) |  | 374 | 1.00 |  | 388 | 1.02 (0.88, 1.18) |  |
| ≥24.0 | 43 | 0.87 (0.61, 1.24) |  | 100 | 1.00 |  | 739 | 0.93 (0.75, 1.15) |  |
| Smoking |  |  |  |  |  |  |  |  | 0.750 |
| Never | 269 | 1.02 (0.86, 1.21) |  | 284 | 1.00 |  | 748 | 0.99 (0.86, 1.14) |  |
| Former or current | 236 | 1.09 (0.89, 1.33) |  | 190 | 1.00 |  | 379 | 0.96 (0.80, 1.15) |  |
| Physical activity^†^ |  |  |  |  |  |  |  |  | 0.730 |
| Low | 187 | 0.92 (0.75, 1.14) |  | 182 | 1.00 |  | 474 | 0.91 (0.77, 1.09) |  |
| Middle | 148 | 1.12 (0.87, 1.43) |  | 135 | 1.00 |  | 340 | 1.02 (0.83, 1.25) |  |
| High | 170 | 1.16 (0.92, 1.45) |  | 157 | 1.00 |  | 313 | 1.01 (0.83, 1.23) |  |
| Intake of supplementary calcium, iron or zinc |  |  |  |  |  |  |  |  | 0.589 |
| Yes | 39 | 1.07 (0.66, 1.74) |  | 33 | 1.00 |  | 97 | 0.91 (0.60, 1.38) |  |
| No | 466 | 1.05 (0.91, 1.20) |  | 441 | 1.00 |  | 1,030 | 0.98 (0.88, 1.10) |  |
| Intake of dairy products |  |  |  |  |  |  |  |  | 0.177 |
| At least once per week | 45 | 0.80 (0.52, 1.21) |  | 51 | 1.00 |  | 119 | 0.75 (0.53, 1.06) |  |
| Less than weekly | 460 | 1.08 (0.94, 1.24) |  | 423 | 1.00 |  | 1,008 | 1.01 (0.90, 1.13) |  |
| Menopausal status (in women^)^ |  |  |  |  |  |  |  |  | 1.000 |
| Premenopausal or perimenopausal | 51 | 0.81 (0.58, 1.14) |  | 104 | 1.00 |  | 261 | 0.99 (0.78, 1.24) |  |
| Postmenopausal | 156 | 1.07 (0.83, 1.36) |  | 121 | 1.00 |  | 363 | 1.04 (0.84, 1.28) |  |

HR indicates hazard ratios; CI confidence interval; MET, metabolic equivalent of task.

Multivariable models were adjusted for the same set of covariates as in model 3 of Table 2.

^*^BMI at age 25 was calculated using self-reported weight at age 25 and measured height at baseline

^†^Physical activity (MET h/day) was categorized using tertile cut-offs by sex (for men: <13.17, 13.17 to <28.25, ≥28.25; for women: <12.33, 12.33 to <23.30, ≥23.30).

Appendix table 12. Association between weight change from age 25 to resurvey and BMD measures among 8,295 men and 13,158 women.

|  | Weight change from age 25 to resurvey, kg | | | | | | | P_trend_ | P_interaction_ |
| --- | --- | --- | --- | --- | --- | --- | --- | --- | --- |
|  | ≤-5.0 | -4.9--2.5 | -2.4-2.4 | 2.5-4.9 | 5.0-9.9 | 10.0-14.9 | ≥15.0 |  |  |
| Participants, n (%) |  |  |  |  |  |  |  |  |  |
| Men | 1,166 (14.1) | 573 (6.9) | 1,444 (17.4) | 817 (9.8) | 1,578 (19.0) | 1,300 (15.7) | 1,417 (17.1) |  |  |
| Women | 1,487 (11.3) | 870 (6.6) | 2,506 (19.0) | 1,525 (11.6) | 2,957 (22.5) | 2,070 (15.7) | 1,743 (13.2) |  |  |
| BUA, dB/MHz |  |  |  |  |  |  |  |  | <0.001 |
| Men | -4.70 (-5.57, -3.83) | -1.86 (-2.92, -0.81) | 0.00 | 0.86 (-0.07, 1.80) | 2.20 (1.42, 2.99) | 2.65 (1.81, 3.49) | 4.24 (3.38, 5.09) | <0.001 |  |
| Women | -4.77 (-5.49, -4.06) | -1.95 (-2.78, -1.12) | 0.00 | 0.97 (0.28, 1.65) | 2.22 (1.64, 2.80) | 3.05 (2.41, 3.69) | 4.78 (4.09, 5.48) | <0.001 |  |
| SOS, m/s |  |  |  |  |  |  |  |  | <0.001 |
| Men | -7.43 (-10.78, -4.09) | 0.06 (-3.98, 4.10) | 0.00 | 3.45 (-0.13, 7.03) | 1.71 (-1.29, 4.72) | -0.89 (-4.09, 2.32) | 0.15 (-3.13, 3.43) | 0.009 |  |
| Women | -3.82 (-6.61, -1.03) | -2.25 (-5.47, 0.97) | 0.00 | -1.92 (-4.59, 0.74) | 4.01 (1.76, 6.25) | 2.94 (0.45, 5.43) | 5.90 (3.19, 8.60) | <0.001 |  |
| SI |  |  |  |  |  |  |  |  | <0.001 |
| Men | -5.20 (-6.58, -3.82) | -1.23 (-2.89, 0.44) | 0.00 | 1.53 (0.06, 3.01) | 1.94 (0.70, 3.19) | 1.52 (0.20, 2.85) | 2.87 (1.51, 4.22) | <0.001 |  |
| Women | -4.24 (-5.33, -3.16) | -1.92 (-3.18, -0.67) | 0.00 | 0.11 (-0.93, 1.15) | 2.59 (1.72, 3.47) | 2.85 (1.88, 3.82) | 4.82 (3.77, 5.88) | <0.001 |  |

BMD, bone mineral density; BUA, broadband ultrasound attenuation; SOS, speed of sound; SI, stiffness index.

All covariates were measured at resurvey. Multivariable models were adjusted for the same set of covariates as in model 3 of Table 3, except for sex. Models in women additionally included menopausal status (premenopausal, perimenopausal, postmenopausal with age at menopause <45, 45-49, ≥50 years, or missing values).
